# Understanding the performance of community health volunteers involved in the delivery of health programmes in underserved areas: a realist synthesis

**DOI:** 10.1186/s13012-017-0554-3

**Published:** 2017-02-16

**Authors:** Gaëlle Vareilles, Jeanine Pommier, Bruno Marchal, Sumit Kane

**Affiliations:** 1 EHESP Rennes, Sorbonne Paris Cité, 9 place de Metz, Grenoble, 38 000 France; 2 CNRS, UMR CRAPE Centre de Recherches sur l’Action Politique en Europe-6051, Paris, France; 3 International Federation of Red Cross and Red Crescent Societies, Community Health and Innovation, Geneva, Switzerland; 4 Department of Public Health, Institute of Tropical Medicine, Antwerp, Belgium; 5 KIT Health, Royal Tropical Institute, Amsterdam, The Netherlands; 6 Gokhale Institute of Politics and Economics, Pune, India

**Keywords:** Motivation, Performance, Community health volunteers, Intervention, Mechanisms, Contexts, Realist synthesis

## Abstract

**Background:**

The recruitment of community health volunteers (CHVs) to support the delivery of health programmes is an established approach in underserved areas and in particular where there are health inequalities due to the scarcity of trained human resources. However, there is a dearth of evidence about what works to improve CHVs’ performance. This review aimed to synthesise existing literature to explain why, how and under which circumstances intervention approaches to improve the performance of CHVs are more likely to be successful.

**Methods:**

We performed a realist synthesis. We identified candidate theories related to our review questions, which then guided the selection, appraisal and analysis of primary studies. Publications of interest dating from 2008 to 2012 were identified by a systematic search in PubMed and IDEAS databases. We considered all study designs that examined the various aspects of CHV performance in the context of formal organisational settings to be eligible and excluded the studies that did not provide explanation about the performance of CHVs neither in the findings nor in the discussion part. Data were arranged according to their reference to context, interventions, outcomes and mechanisms in order to identify the interaction between them. The synthesis of data allowed us to determine explanatory patterns within or across the studies.

**Results:**

We identified broad intervention approaches within the 23 papers included in the review: positioning of the CHV within the community, establishment of clear roles, provision of skill-based and ongoing training, incentives, supervision and logistical support for task distribution and implementation. The findings provided information regarding which mechanisms (self-esteem, sense of duty, self-efficacy, sense of being fairly treated) to target when implementing such approaches, and which contextual factors (stable and supportive cultural, political and social context and intervention closely linked to local health services) create the most favourable conditions for these mechanisms to occur, ultimately contributing to CHVs’ better performance. Four main explanatory patterns around these mechanisms emerged as being fundamental to better performance.

**Conclusions:**

The patterns identified, combined with the designers’ and other stakeholders’ assumptions on how such interventions are expected to work, can be tested by empirical studies in order to provide useful information to be used by programme implementers, policymakers, donors and the community.

**Electronic supplementary material:**

The online version of this article (doi:10.1186/s13012-017-0554-3) contains supplementary material, which is available to authorized users.

## Introduction

Globally, there is a dire shortage of healthcare workers. In many developing countries, the health workforce crisis is a pressing issue that is a major obstacle to achieving the health-related Millennium Development Goals [1, 2]. One of the strategies tried by many countries to address this problem includes task shifting to less qualified health workers. Community health volunteers (CHVs), who are trained lay volunteers and provide health services in their local communities, are an important part of a task shifting strategy and may help address health worker shortages.

Experience has shown that CHVs represent important and unique resources. Trained CHVs can deliver crucial and culturally sensitive health messages, empower individuals to make informed decisions and increase local access to life-saving curative measures [3–7]. However, the performance of CHVs is being questioned by the public health community: high attrition rates and poor quality services have been reported in many programmes involving CHVs [8–11]. Others report how CHVs’ ability to deliver is contingent on the scope of their work and workload [12, 13]. In their guidelines on task shifting, the World Health Organization acknowledges the contribution of short-term or part-time volunteers but states that “there is virtually no evidence that volunteerism can be sustained for long periods” [14, 15].

The existing studies on the performance of volunteers highlight the importance of individual motivation [16, 17]. Indeed, performance can be perceived as a behavioural consequence, such as task performance, task persistence, attendance and organisational citizenship behaviour, of the level of motivation, itself influenced by working conditions and other contextual factors [18–20]. These factors include both financial and non-financial incentives, such as training, management practises and community support [7, 9, 21, 22].In general, literature on the performance of volunteers is sparse and the debate surrounding what motivates CHVs to perform well is still salient. One much debated issue concerns the appropriate mix of incentives to motivate volunteers and whether the motivational drivers of non-salaried volunteers differ from those of salaried health workers. Some studies indicate the importance of financial incentives (payment to cover out-of-pocket expenses, micro credit funds) or non-financial incentives with financial value (free health care, bicycles or future job opportunities) as a vital strategy to improve the level of activity of CHVs. Indeed, CHVs are likely to be drawn from poor communities and could see volunteering as income-generating [23, 24]. On the other hand, some authors argue that a number of non-financial incentives (recognition, positive feedback, social prestige) may not only be sufficient but actually be more effective in improving performance [25, 26] and that the evidence used to support the non-sustainability of CHVs’ interventions refers to situations where attrition rates are tied to frustration from not receiving promised payment rather than to volunteerism itself [26].

Systematic reviews on the effectiveness of community health interventions delivered by non-professional health workers underline difficulties in drawing consistent conclusions, and authors raise questions about the applicability of findings to different settings. Little is known about how such interventions bring about change, the role a lay health worker can assume and the reasons behind successes and failures [5, 6, 27–29].

Yet, CHVs’ performance and its determinants vary a great deal depending on the context [9, 14, 26, 30], and most of the literature has examined the topic by focusing on barriers or incentives to performance. Since CHV interventions are complex and embedded in complex health and social systems and contexts, a one-size-fits-all scheme for sustaining ‘high performance’ of CHVs around the world is unlikely to succeed. More research is needed to better understand how different models and combinations of incentives influence CHVs’ motivation, retention and performance, and in which particular conditions these happen.

### Research aim, objectives

The aim is to synthesise existing literature to explain why, how and under which circumstances intervention to improve CHVs’ performance is more likely to be successful.

The specific objectives are to:

- Document the interventions implemented to develop good performance in CHVs
- Identify the mechanisms that explain how the interventions contribute to CHVs’ performance
- Investigate the circumstances that trigger these mechanisms
- Develop plausible explanations on why and how the interventions contribute (or not) to improve CHVs’ performance

This review is also part of the International Federation of Red Cross and Red Crescent Societies’ (IFRC) research project that aims to better understand how Red Cross and Red Crescent volunteer motivation can be enhanced by appropriate approaches. The IFRC reaches 150 million people annually through 190 member National Societies and 17 million volunteers worldwide. The National Societies implement programmes in order to develop a pool of capable volunteers who can improve health in underserved communities.

For the purpose of this synthesis, we then consider CHVs as health workers who provide services within a formal structure. CHVs often complement professional service delivery but can also be important agents of social change by detecting unmet needs in society. They do not have a formal medical background but are trained and provide health services in their local communities. They do not receive a regular salary but may receive other benefits.

## Methods

### The methodological approach

We performed a realist synthesis. Realist synthesis is a theory-driven, qualitative approach to synthesise qualitative, quantitative and mixed methods research evidence [31]. By drawing on broader theoretical insights and using evidence, it offers the potential to develop the intervention theory—hypotheses on how, why and in what circumstances an intervention may be successful—that can be tested in subsequent empirical studies. To uncover such underlying theory (or theories) of intervention, realist synthesis examines the interaction between the intervention, the context (C) within which it is applied, the mechanism (M) that describes how the actors use the resources available to them and the outcome (O) in a sample of primary studies [32]. These interactions are formally articulated as context-mechanism-outcome (CMO) configurations. As CMOs emerge and insights accumulate, the result can attain the level of middle-range theory (MRT). The MRT operates at a slightly higher level of abstraction, being a theory in between grand universal theories and the more specific programme theory that explains the results of a particular intervention.

The key concepts as well as the key terms of the realist approach used for this study are illustrated in Table 1.

**Table 1:** Key concepts and key terms of the realist approach to evidence synthesis (adapted from Robert et al. [85], Dalkin et al. [86] and Pearson et al. [87])

Mechanism	Element of the reasoning of the actor facing an intervention (beliefs, values, desires and cognitive processes). A mechanism (1) is generally hidden, (2) is sensitive to context variations and (3) produces outcomes.
CMO configuration	Conceptual tool to link the elements of context, mechanisms and outcomes of an intervention
Programme theory	Set of hypotheses that explain how and why the intervention is expected to produce outcomes. It can be broken down in the form of one or more CMO configurations.
Middle-range theory	Level of theoretical abstraction that provides an explanation of semi-regularities in the CMO interactions of a set of interventions
Demi-regularity	A demi-regularity is a semi-predictable pattern or pathway of programme functioning
Juxtaposition, reconciling, adjudication and consolidation of sources of evidence and situating sources of evidence	-Juxtapose, to place two or more things (evidence fragments) together, especially in order to suggest a link between them or emphasise the contrast between them -Reconcile, to make two or more apparently conflicting things (evidence fragments) consistent or compatible -Adjudicate, to make a judgement about methodological quality or applicability in this instance and account for this judgement based on findings from the use of the critical appraisal tool, or an explicit argument about why a piece of evidence was not applicable -Consolidate, to bring together. In a realist synthesis, ‘to bring together into a more coherent whole’ -Situate, to place something (a piece or pieces of evidence) in a context or set of circumstances and show the connections (between it/them and other evidence fragments)

The choice of the synthesis method was made according to the research questions and the key features of the intervention under study [33, 34]. We consider realist synthesis an appropriate choice as the use of theory and its methodological tools helps to capture the complexity of human resources by including the context in the analysis. The realist perspective allows for a better understanding of how strategies to improve CHVs’ performance are supposed to work and what contextual factors affect the outcome [35]. Conventional systematic reviews methodologically limit the development of explanations of the changes brought by an intervention.

This synthesis seeks to identify key mechanisms at play in driving CHVs’ performance. These mechanisms are triggered by context-intervention dynamics, and therefore, mechanisms are the result of complex interaction between the intervention approaches and CHVs.

We followed the steps proposed by Pawson in conducting this realist review [36]: (1) identifying potential theories, (2) searching for evidence, (3) appraising primary studies, (4) extracting data, (5) analysing and synthesising evidence. The main differences compared to a traditional systematic review include a purposive and theoretically driven search and appraisal of evidence with the aim of refining theories, the possibility of including multiple types of information and an iterative process. The ultimate goal is to explain why and under which conditions interventions worked [32, 37].

The present article is structured around these five steps and uses the realist and meta-narrative evidence synthesis (RAMESES) publication standards for reporting realist synthesis [38].

### Step 1—identifying potential theories

- Discussion with key stakeholders

We consulted key experts on volunteering at the IFRC headquarters in Geneva to develop insights on why some approaches improve CHVs’ performance more than others. Two meetings were organised, and issues regarding the performance of the Red Cross and Red Crescent volunteers were debated. The key elements of these discussions were then explored through the literature.

(b) Identifying candidate theories

We performed a scoping review using a ‘snowball’ technique in order to map the existing social science theories related to our research objectives. For this, we undertook a review of a wide range of evaluation, management, organisational behaviour and work motivation literature. The theories that were selected are provided in Additional file 1.

(c) How these theories inform our review questions

In general, performance is influenced by each CHV’s individual cognitive (knowledge, skill acquisition) and affective attributes (self-efficacy, ability to master challenges, self-esteem, being close to other people, satisfaction with the work, emotional attachment to the organisation), the intervention approaches and the organisational context in which the CHV operates as well as the broader social, cultural and political context surrounding the CHV.

More specifically, the learning process of the CHV may improve his/her competences. This process is influenced by factors within the organisational environment and, more specifically, related to the content of training, learning methods, feedback from others and the personal experience of the CHVs in dealing with a problem. The CHV’s behaviour and work effort is influenced by the degree of motivation and metacognition, which are themselves influenced by the background and past experience of the CHV, the community setting, the social, cultural and political context of the organisational setting and the context of the intervention.

**Fig. 1:**
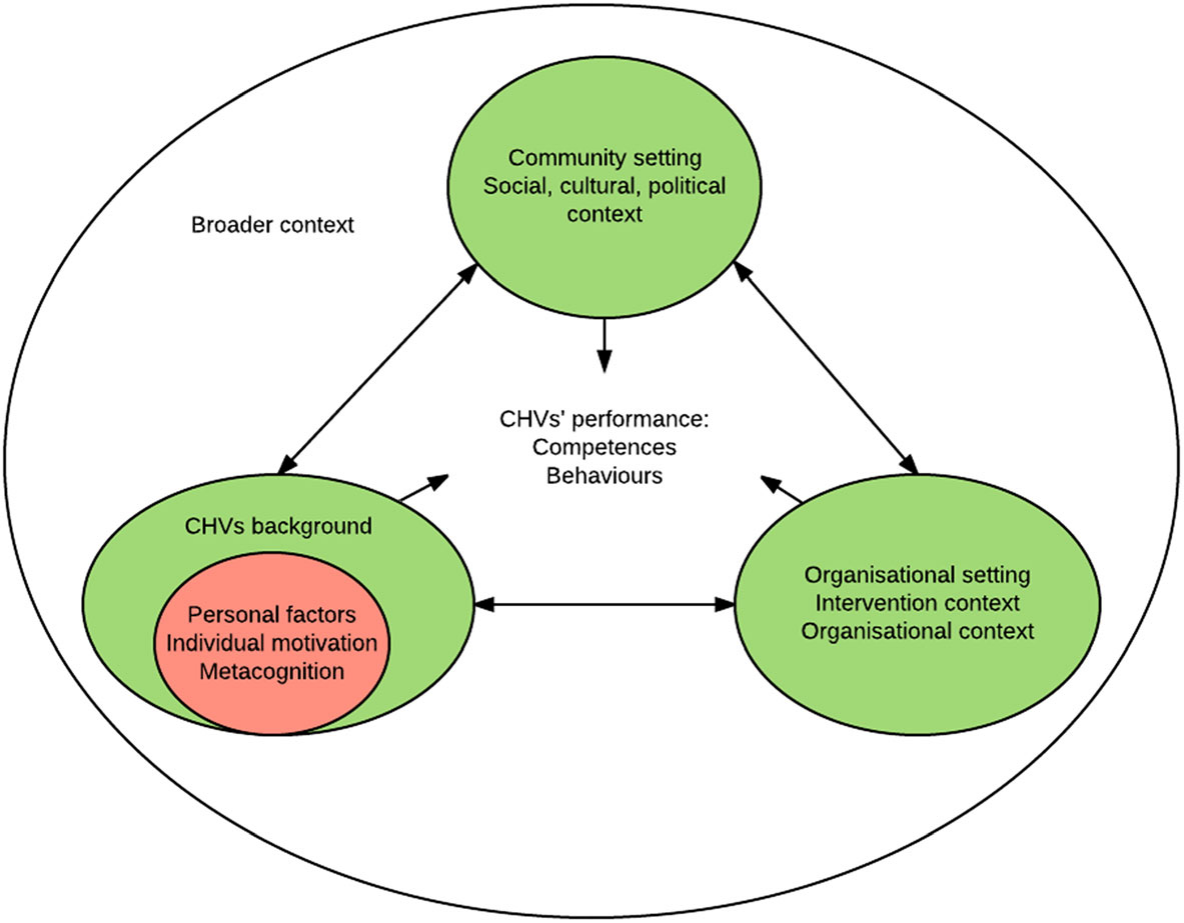
Flow of the diverse influences on CHVs performance

The diverse influences on CHVs’ performance, informed by the theoretical landscape, are illustrated in Fig. 1.

### Step 2—searching for evidence

Following our scoping review that identified candidate theories, we undertook a search for primary evidence. The search was guided by our research questions and by the candidate theories that were identified in the scoping review. We considered all study designs to be eligible.

The search was made in PubMed and IDEAS databases from 2008 to 2012, and the keywords used were community health volunteers, community health workers, lay health workers, and task shifting. This allowed us to identify a range of primary studies dealing with our initial understanding of how and under what circumstances CHVs may perform well.

We decided to focus our search strategy this way because of the huge number of primary studies and some capacity constraints. Indeed, the process of selecting the number of articles that are potentially eligible for inclusion can only occur as the data sources are analysed in detail. As an example, we initially planned to perform a search in various databases and to review the existing evidence published since 2000. However, to provide an example, a search from 2000 to 2014 in PubMed using community health workers, community health volunteers as keyword provides 3339 items. Some articles can be easily excluded on the title or on the abstract. However, an approximation of 1000 articles remain to be read in order to assess whether they could contribute to find explanation on why and under which conditions the interventions worked.

### Step 3—appraising primary studies

The relevance of the studies was assessed according to the focus of the review and how each study addressed the research questions. Decisions about whether each study could contribute to identifying CMO configurations and whether the method used to generate these CMO configurations is credible were arrived through discussion in the review group.

The specific inclusion and exclusion criteria that guided the selection and appraisal of documents are presented in Table 2.

**Table 2:** Specific inclusion and exclusion criteria

Inclusion criteria	Exclusion criteria
Published articles examining the various aspects of CHVs’ performance in the context of community health interventions that target poor populations with unmet health needs and where CHVs: • are engaged on a voluntary basis in a formal organisational setting (with professional engagement in recruitment, training or support) • do not have a formal medical background • are recognised as members of the community	Published articles examining volunteers who undertake support and education activities informally without professional direction, such as true natural helpers and expert patients Published articles not reporting on the outcome of interest (CHVs’ performance) Published articles not addressing the research questions and/or not contributing to the development of candidate theories, refinement and/or clarification (CMOs configuration development)

### Step 4—extracting data

The documents included in the review were compiled in Excel. The variables extracted were (1) authors, title of the study and year of publication; (2) study objective and outcomes; (3) intervention characteristics and objectives; (4) prior assumptions reported by the authors; (5) mechanisms gleaned by reader and/or reported by authors; (6) reported/observed actual outcome(s); (7) contextual factors reported by authors to be of influence on CHVs’ performance and (8) process of intervention implementation/stakeholder involvement reported by authors to be of influence on CHVs’ performance.

### Step 5—analysing and synthesising evidence

For the sake of clarity, we report on the process of analysis and synthesis in two stages, although in practise, this was a reiterative process guided by the review questions. An overview of the activities, the analytical process and the use of candidate theories, is provided in Table 3.

**Table 3:** Overview of the activities, analytical process and use of candidate theories according to the two stages of the analysis and synthesis process

Stage of the analysis and synthesis	Activities	Analytical process	Use of candidate theories
Evidence drawn from each primary study	Identification of the link between context, mechanism and outcomes Specification of CMO(s) configuration	Mix of abductive and retroductive reasoning Constant comparative analysis	To guide the analytical process
Synthesis of data within or across primary studies	Identification of potential explanatory patterns	Juxtaposition, reconciling, adjudication, consolidation of sources of evidence Situating sources of evidences	To explain at a higher level the explanatory patterns that emerged across the studies

First, using a mix of abductive and retroductive analytical processes, each primary study was examined for evidence based on how it supports, refutes or refines the candidate theories identified in the first step [39, 40]. The candidate theories guided the identification of emerging themes through the process of constant comparative analysis [41]. From the disconnected variables extracted and compiled in Excel, a summary table was developed from each paper under review. The main themes were arranged according to their reference to context, interventions, outcomes and mechanisms in order to identify the interaction between them. The tables were then reviewed by all the authors, and any differences of opinion were resolved by consultation and discussion of the paper. For each paper, we specified CMO configuration(s) and drafted a narrative synthesis.

Secondly, through a process of reasoning, including juxtaposing, reconciling, adjudicating, consolidating and situating all sources of evidence [36], we synthesised the data from the included papers in order to identify potential patterns. While doing so, insights from substantive social science theories were used to explain at a higher level the explanatory patterns that emerged across the studies. This stage consisted in articulating explanations for intervention and outcomes based on the papers under review. To this end, mechanisms were compared across different contexts to assess if they consistently produced similar outcomes. Moreover, in adjudicating between different sources, we were careful to use the findings of the critical appraisal in relation to the relevant aspects or insights from a study rather than judging the validity of each study as a whole. If our initial critical appraisal was unable to support a judgement about a particular piece of evidence, we returned to the original source so that a bespoke appraisal incorporating rigour and relevance could be made. The research team convened on a regular basis to discuss the patterns that emerged and their degree of fit with candidate theories. Finally, we discussed the findings in view of the larger worker motivation and performance literature and, methodologically, the literature on realist synthesis.

## Results

**Fig. 2:**
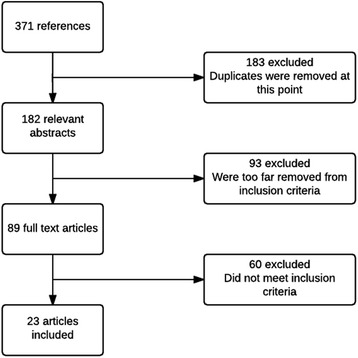
Flow diagram for search process and study selection

The number of studies retrieved and the steps undertaken are detailed in Fig. 2.

Additional file 2 summarises the studies, the methods used, the context, the intervention, the mechanisms triggered and the outcomes.

### Study characteristics

Eleven qualitative studies [26, 30, 42–50], one randomised controlled trial (RCT) [51], one cluster RCT [52], six cross-sectional studies [53–58], three case-control studies [24, 59, 60] and one cohort study [61] were reviewed. The studies reported on CHVs’ involvement in a wide range of health service delivery. Six studies focused on CHV interventions for promoting health/educational activities that did not specifically involve service delivery at homes of beneficiaries. Ten studies [26, 30, 44, 45, 47, 50, 54–57] reported on CHV interventions for delivering home-based care. Four studies focused on HIV/AIDS management in facilities or HIV counselling centres [48, 52, 58, 61]. All the studies targeted poor communities with unmet health needs (existing physical, financial and educational barriers to health, with no current culturally appropriate health interventions).

### Interventions implemented to develop a pool of well-performing CHVs in the primary studies

The interventions discussed in the papers consisted of different intervention approaches. We identified a number of broad categories:

- Approaches involving the positioning of the CHV within the beneficiary community through explicit selection of CHVs by the community and positioning the CHV as a role model
- Training of CHVs that involves provision of knowledge and skill-based training, including ongoing or refresher training
- Delivery of compensation and recognition for the work of CHVs through the provision of monetary and non-monetary incentives
- Establishment of clear roles (roles made clear to CHVs, other health workers and beneficiary communities), supervision and logistical support for task distribution and implementation

In 17 papers, the interventions were implemented by local governmental agencies [26, 30, 42, 43, 46–52, 54–58, 60], and for some of these agencies, local non-governmental organisations (NGOs) provided support [26, 30, 57]. In one study, the intervention was implemented through public-private partnerships [50]. Local NGOs were the main implementing organisation in the other six studies [24, 44, 45, 53, 59, 61].

### Outcomes of the interventions in the primary studies

The studies reported a variety of positive primary outcomes at the level of CHVs, such as satisfactory competence and practises, quality of services, availability, work effort and active participation in the programme, ability to exercise leadership, community engagement and mobilisation, improvement in access to health care, reduction of patient waiting time and the workload of formal health care workers, retention, satisfaction and well-being. Nine studies reported and discussed negative outcomes around dissatisfaction, attrition, reduction of involvement and effort, lack of community mobilisation, poor leadership and sustainability of the programme and inappropriate health practises [30, 44, 45, 48, 50, 53, 54, 57, 60]. Most of the studies included were not set up to assess or explicitly reflect upon the relations between these specific outcomes and the mechanisms gleaned by us as reviewers.

### Explanatory patterns that emerged across the studies: CMO configurations

We identified a number of demi-regularities across the studies as being fundamental in generating positive outcomes. They can be structured around four families of mechanisms: (a) positive links between community members and CHVs, (b) mastering new tasks and ability to solve problems (capability), (c) CHVs’ feeling of recognition by the organisation for the work done and (d) role identification and recognition across the health system. These demi-regularities are presented below.

- Positive links between community members and CHVs

In a context where community members value volunteering as a morally good behaviour and when a socially desirable outcome or achievement exists (such as on-the-job certificated training and opportunities for personal growth), an intervention which creates opportunities for developing close, trusting ties between community members and CHVs through providing opportunities to meet this socially desirable outcome or achievement and through clear positioning of the CHVs within the community can produce positive outcomes.

We identified the mechanisms of self-esteem/sense of pride/sense of duty/sense of community recognition as a driver of CHVs. We noticed that success is facilitated if there is a stable and supportive social, material and political context and a strong history of social organisation. Since such interventions operate in a dynamic social system, early visible positive results among community members facilitated positive outcomes through feedback loops.

Negative results also demonstrated the explaining power of this demi-regularity [24, 44, 59, 60]. The volunteers were less likely to be active, remain engaged, take additional responsibilities, exercise leadership or mobilise and organise community responses (outcomes), if there was an unsupportive cultural, social and political context and if the CHVs themselves did not have a prior positive reputation within the local community, particularly among the community’s leaders. In such unfavourable contexts, the mechanisms identified did not have sufficient opportunity to fire.

(b) CHVs mastering new tasks and ability to solve problems (capability)

When few educational opportunities exist, a community-based, volunteer-oriented health intervention that provides (1) skill-based and ongoing training, (2) supportive supervision and follow-up and (3) resources adapted to local context and logistical support to perform the work triggered among the CHVs a feeling of their ability to perform the task, a sense of self-efficacy and self-confidence.

It also emerged from the papers that early visible results among community members and positive community feedback reinforces self-efficacy/self-confidence among CHVs; they felt that the health of their community had improved as a result of their activities. The positive experience of the CHVs and the ability to self-organise activities reinforce self-efficacy and ownership over time. These mechanisms contributed to positive outcomes.

In some studies, the mechanisms of self-efficacy and self-confidence were not triggered, and the CHVs had the feeling that they could not live up to the expectations of the communities. This is more likely to happen in remote rural areas requiring long travel time to reach beneficiaries’ homes and the health centre if the intervention is implemented without investment in strengthening the health system, if the community expresses an inability to deal with a disease or if the trust by the community in the health services is lost. In such cases, the satisfaction of the CHVs, their involvement and the quality of their services are undermined [30, 48, 54, 57].

(c) CHVs’ feeling of recognition by the organisation for the work done

We found that providing adequate support to CHVs based on fairness and equity triggered a feeling of being appreciated for the work done and of being valued and thereby contributed to positive outcomes. The kind of incentives adapted or not to the local cultural, social and economic context influenced these outcomes.

More specifically, in the context of poor communities with few opportunities to increase family income and when the involvement of the CHVs in the programme costs them time and money, the provision of incentives with a financial value, such as meals, stipends, free access to health care and micro credit funds meaningfully recognises the CHVs for their efforts and triggers a sense of being fairly treated; this feeling results in a high level of accountability to perform. CHVs feel satisfied with the nature of benefits that they and their immediate family members are receiving, and this contributes to better performance (better retention and active participation in the core activities of the programme).

Furthermore, providing non-monetary incentives (including moral and verbal support, tokens of appreciation, recognition signs and events, on-the-job training, certificates and equipment) as well as a safe- and friendly-working environment triggers the perception of organisational support, recognition for CHVs’ contribution and the feeling that their expectations are fulfilled. It also triggers the feeling of being part of the organisation. This contributes to positive outcomes, such as better retention and task performance.

The adaptation of incentives to the local context influences the outcomes. In the context of urban areas where competing opportunities exist, community-based health volunteering interventions that aimed at involving younger, aspirational CHVs resulted in higher attrition rates if they did not help them meet their needs/initial expectations.

According to some studies, financial incentives can undermine other incentives and the moral status of volunteering, especially when volunteering is considered culturally as a morally good behaviour and when paid government workers are not respected [26, 46]. However, in a context of high poverty, where earnings are important for sustaining a family, monetary incentives that are easily distinguishable from a salary triggered a feeling of acknowledgment and recognition for their contribution. In such cases, positive outcomes such as high retention, involvement in the core activities of the intervention and satisfaction among the CHVs were observed [24, 26, 46, 54, 56].

(d) Role identification and recognition across the health system

In the context where CHVs are answerable to the health service personnel, a socially desirable community-based health volunteer intervention with (1) specific role definitions, a legal framework and precise procedures for task shifting, (2) support for CHVs in the form of follow-up training and regular supervision from the formal health staff and (3) logistical support can trigger among the trained CHVs a sense of legitimacy, a sense of being valued by health authorities, a deep sense of commitment to their role and a professional identity around this role.

These mechanisms seem to contribute to positive outcomes and were more likely to be triggered when CHVs were well integrated into the operations of their facilities, were acceptable as providers and readily used by clients and when the involvement of the CHVs did not further burden clinical staff.

However, in the context of a weak, dysfunctional and unregulated health system, the involvement of a new cadre of health workers in the public domain, such as CHVs, did not always allow these mechanisms to fire. In that case, it contributed to poor performance, dissatisfaction and high dropout. In the study reported by Schneider [50], the interventions were implemented through public-private partnerships, and this triggered among the CHVs a feeling of being an exploited labour force and of uncertainty and distrust with regard to the formal health staff. This was mainly due to the lack of integration between governmental and non-governmental agencies and the absence of a broader strategy to strengthen supply, management and deployment of human resource for health. In other studies, intervention approaches that lacked clear role descriptions and career progression triggered among CHVs a sense of difficulty to prove their legitimacy and to develop their identity. Their expectations were not met [48, 59] and distrust with regard to the formal health staff ensued (mechanisms) [48].

## Discussion

### Linking CMO configurations and theories

The concepts we identified in the included study map across many different theories (Additional file 1).

A close link between community members and CHVs was identified in this review as being one of the fundamental elements in generating positive outcomes. CHV organisations are embedded in a community context, which is both the target of the organisations and a source of influence on the CHVs’ individual motivation and attitudes. As mentioned by Strachan et al. when referring to the social identity approach [62, 63], volunteers’ behaviour is subject to perceived group social norms and to what is seen to be in the group’s interests. We found furthermore, in line with Hustinx and Lammertyn who studied the nature of volunteering through the lens of sociological modernisation theories [64], that a stable and supportive social and political context and a strong history of social organisation can be a base for collective volunteerism, in which dedication to the common good is a highly esteemed asset and a sense of duty or responsibility to a local community is a strong motivational driver. Finally, most of the interventions reported in the studies we reviewed operate within the context of community change. According to the existing theories on community change [65, 66], CHVs can exercise leadership, promote critical consciousness and contribute to developing the potential for collective actions. Our synthesis showed that a stable cultural, social and political environment as well as the positive reputation of CHVs within the local community were important contextual factors for these outcomes to be achieved.

CHVs’ feeling of self-efficacy and their ability to overcome challenges were keys in determining positive outcomes. As predicted by Bandura’s social cognitive theory where self-efficacy is the major concept [67], we found that CHVs are more likely to mobilise efforts for service delivery if they believe it is attainable through their endeavours. More specifically, the extent to which CHVs feel their service has met community members’ expectations as well as fulfilled their personal needs, their perception of their work and their perceptions of other people’s reactions to their work are all key factors that can trigger a sense of self-efficacy and self-confidence. This has also been described by Omoto and Snyder in their volunteer process model that focuses, among other things, on how people feel in themselves and how they feel about the organisational context during their initial experiences as a volunteer [68].

The volunteer work reported by the papers we reviewed is situated in a formal organisational setting. Regarding the delivery of incentives to CHVs, our review confirms that if various forms of volunteering exist in developing countries, individuals are generally interested in both the private and public benefits of volunteering. This is in agreement with the mixed public/private models of volunteering suggested by Schiff and others [69, 70]. We found that the possibility of improving the performance of the CHVs is a function of the degree to which the incentives are perceived to satisfy the CHVs’ needs, these needs being themselves influenced by the social or contextual factors. For instance, among the studies reviewed and in the context of high poverty, monetary incentives or non-monetary incentives that have financial consequences contributed to positive outcomes and did not undermine other incentives because they encouraged social recognition, triggered a sense of fair treatment and fulfilment of basic needs. The results are consistent with need satisfaction theories, where the focus has been on finding the right match between incentives, the individuals and their context [26, 71–73]. Responding to volunteers’ needs is in accordance with social exchange and equity theories where commitment and good performance occur as individuals and group interests are achieved through reciprocal exchange, fairness and appreciation [74, 75].

Our results are in line with the consistency theory [76] as well as organisational commitment [77] and social exchange theory [74]. According to these theories, the culture of an organisation, its structure, leadership, vision and mission and employee management all have a direct influence on the perceptions of organisational support, trust with regard to the organisation, sense of legitimacy and hence on the commitment and motivation of the employees.

Globally, our results showed that the CHVs as human beings have an inherent need for care, respect, trust, recognition and support. When these needs are satisfied, CHVs are often encouraged to work harder and perform better. The mechanisms we identified as being important for CHV performance are also in line with, and build upon, the findings from earlier realist analysis examining the performance of those working closely with communities [78–81]. Therefore, taking a holistic view based on humanistic values in dealing with the CHVs seems relevant. Because of this, the Self Determination Theory (SDT) identified in step 1 (see Additional file 1) could provide an appropriate framework for the study of CHV motivation and behaviour in the context of an organisational setting. According to this framework, humans actively pursue fulfilment of their basic psychological needs [82], and the degree of satisfaction of these needs supports different forms of motivated or regulated behaviour in a variety of contexts and situations [83].

### Practical implications and limitations

The wide range of study designs included in this realist synthesis offered a fair amount of information about CHV-based interventions, contexts and mechanisms through which performance of CHVs can be explained. We reviewed the studies against existing broad theories, identified regular patterns in CHVs’ dispositions to performance, and attained the level of MRT. With the identification of mechanisms, the context becomes central in explaining the performance of the CHVs: we know from the literature that CHVs’ performance and its determinants (such as initial training, supervision, level of education, types of incentives, etc.) strongly depend on the context. This synthesis suggests that many contextual factors influence outcomes. For example, in the context of high poverty, CHVs cannot feel adequately recognised without monetary incentives, or without non-monetary incentives that have financial value. In the contexts where there are few opportunities for stable social relationships, or supportive norms to develop, CHVs cannot feel sufficiently respected or valued and, as a result, cannot expect to improve their social status by volunteering.

Recommendation for CHV interventions should therefore emphasise the need to take into consideration CHVs’ perceptions of these mechanisms and adapt interventions to the local context. The insights from this realist review have implications for policymakers and programmers interested in leveraging the potential of community health volunteers. Based on the evidence reviewed, effective approaches, including approaches which involve clear positioning of the CHV within the community (examples from current evidence include establishment of a local health committee involving CHVs and creating processes for community feedback and for CHVs to play a key role in such processes), approaches which involve explicitly creating opportunities to sign to the volunteers that they are valued and recognised (e.g. through organising felicitation and/or graduation ceremonies…) and approaches which entail clarity of roles, provision of skill-based and ongoing training, incentives adapted to local context, good supervision and logistical support, can trigger mechanisms (sense of self-esteem, sense of duty, sense of self-efficacy, sense of being fairly treated if the organisation is responsive to the local needs, sense of recognition and legitimacy) which are likely to translate into effective, meaningful and sustained volunteer engagement. These intervention approaches can trigger these mechanisms in a stable and supportive cultural, political and social context and where the intervention is closely linked to local health services. That said, these intervention approaches may or may not trigger the said mechanisms in contexts which are unstable and not supportive to the functioning of CVs. More specifically, within the context of the Red Cross Red Crescent Movement, the results of this synthesis informed the development of the programme theory for the volunteers involved in delivering health services in stable and supportive contexts [84]. Indeed, the programme theory that explains how and why the volunteerism intervention is expected to produce outcomes should be developed ideally both from the literature and the voice of the main stakeholders (programme designers, managers and volunteers). Drawing on a programme theory that is sensitive to context and tested by empirical studies is more likely to lead to the development of effective interventions when aiming to improve motivation and performance of CHVs [80].

This synthesis has some limitations. Since our review was limited to published articles, there was potentially a bias toward including articles that found significant results supporting CHV performance. Studies that found non-significant or negative results of CHV interventions might have provided additional insights regarding mechanisms and contextual factors that hinder CHV performance. The other limitations are inherent to the nature of realist approach. Firstly, the amount of information to be reviewed can be very large and the compilation of the analysis difficult. We attempted to make the findings clearer by presenting them around the main families of mechanisms in the results and discussion sections. Secondly, it is possible that some contextual factors not consistently reported in the evaluations could be relevant. Third, the CMOs and the patterns identified in this review should be considered as provisional and need to be tested in different contexts and in relation to other conditions. Finally, there may have been outcomes that were not sufficiently explored due to methodological constraints. Different performance outcomes were generally reported in the studies reviewed and it was difficult during the analysis to identify the link between more specific outcomes and the mechanisms.

## Conclusions

This realist synthesis aimed to explain through which mechanisms and under which contextual factors intervention to improve CHVs’ performance is more likely to be successful. The findings provide valuable information regarding which mechanisms to target when implementing the intervention and which contextual factors create the most favourable conditions for these mechanisms to occur, ultimately contributing to CHVs’ better performance. We identified four main explanatory patterns around the mechanisms identified that inform future research. These patterns, combined with the stakeholders’ assumptions (programme managers, designers and volunteers) on how such interventions are expected to work should be tested by empirical studies in order to provide more detailed, useful information to be used by programme implementers, policymakers, donors and the community.

## Additional files

Additional file 1: Selected theories relevant to study volunteering and CHVs’ performance. (DOCX 36 kb)
Additional file 2: Overview of the methodology used, contextual factors identified, the interventions, the mechanisms triggered and the outcomes in the selected studies. (PDF 502 kb)

## Abbreviations

CHV: Community health volunteer
CMO: Context-mechanism-output configuration
IFRC: International Federation of Red Cross and Red Crescent Societies
MRT: Middle-range theory
NGO: Non-governmental organisation
RCT: Randomised controlled trial
