# Supplementary material for: Understanding the performance of community health volunteers involved in the delivery of health programmes in underserved areas: a realist synthesis
Source: Implement Sci. 2017 Feb 16;12:22. doi: 10.1186/s13012-017-0554-3 (PMC5314678; doi:10.1186/s13012-017-0554-3)
Supplement: Additional file 1: — Selected theories relevant to study volunteering and CHVs’ performance. (DOCX 36 kb) [file 13012_2017_554_MOESM1_ESM.docx]

Additional File 1: Selected theories relevant to study volunteering and CHVs’ performance

| Theory | Theoretical premise | Intervention informed by the theories |
| --- | --- | --- |
| The social identity approach [1,2] | Community influences social norms and priority needs of volunteers  When a group identity is relevant to a volunteer and (s)he identifies as a group member, volunteers’ behaviour becomes subject to perceived group social norms and what is seen to be in the group’s interest | Stimulate a feeling of shared experiences and collective identity among volunteers  Promote the value of achieving CHV programme goals to the CHV collective  Clearly communicate the actions that lead to good and appropriate performance and promote the link between this performance and the distinctiveness of the CHV collective |
| Empowerment theory [3]  Participatory education theory [4,5] | Volunteering promotes individual storytelling, awareness and eventually critical consciousness, and offers potential for collective action  The horizontal process of peers (equals) talking among themselves and determining a course of action is key to the impact of peer education on behaviour change | Intensive support of volunteers in their development of leadership experience and skills  Assist volunteers in developing productive linkages within the community (community leaders, development committees, advisory groups, coordination and oversight bodies, etc.) and in promoting participatory activities in the community |
| Adult and social learning theory [8]or social cognitive theory [6,7]  Key concept: self-efficacy [9] | Change occurs when an individual has personal experience with a problem and helps develop the solution  As people engage in response behaviours, they develop a sense of self-efficacy from their experience  The more they develop a sense of self-efficacy, the more they will take on new responsibilities | Use methods, materials and resources for the volunteers during training and programme implementation that are adapted to the local context  Develop learning objectives based on the learner’s needs, interests, and skill levels  Use training that is skill based and problem solving oriented  Setting small and achievable goals  Provide positive feedback on a specific task and reward for a task well done |
| Knowledge transfer theory [10,11] | Assumes that people engage in a set of rational activities, weigh all alternatives, and carefully scrutinise available evidence  Knowledge can enhance practitioners’ autonomy, expand their role  People with fewer qualifications may adhere more strictly to simple clinical guidelines  Locally developed knowledge is more acceptable | Provide simple tools to perform the tasks and adapted to local context  Develop clear and simple protocols with pre-agreed definitions of tasks, |
| Crowding theory [12] | Extrinsic goals coming from outside the self, including salaries, can crowd out intrinsic motivation | Appropriate incentives: align context-specific expectations of community health volunteers, programme managers and policy makers for low attrition and high performance to be achieved |
| Intrinsic motivation theory [13]  Public good model [14]  Private benefits model [15]  Impure altruist [16,17] | People choose humanitarian aid work not because it provides them with material extrinsic rewards, but because it creates immaterial intrinsic rewards  Pure altruism: volunteers are driven by a desire for the well-being of the recipients  Volunteers receive private benefits such as joy from the very act of volunteering  Individuals are interested in both private and public benefits of volunteering | Compensate for humanitarian aid work with non-financial incentives |
| Maslow’s hierarchy of human needs theory of self-actualization and self- transcendence [18] | Accounting for lower needs can improve recruitment and performance  Volunteerism is an activity that allows people to experience satisfaction of their psychological needs | Assist volunteers to make sure their physical and safety needs are met  Allow for participative approaches, stimulate interdependence among volunteers, identification with work groups and space for volunteers to meet their peers in order to develop committed volunteers |
| Self-determination theory [19] | Conditions supporting the individual’s experience of autonomy, competence and relatedness foster the most volitional and high quality forms of motivation and engagement for activities  Satisfaction of psychological needs will be associated with more effective performance and well-being | Match the task to the volunteer’s functional motives  Allow the volunteer to participate in the development of a programme  Provide supportive supervision  Allow interdependence among volunteers and identification with work groups  Provide in-kind rewards, appropriate material and task support |
| The functional approach [20] | When volunteers’ important motivations for service are paired with features of the environment that allow them to actualise these motivations, then volunteers will be more satisfied and more likely to continue volunteering in the future | Provide appropriate incentives  Match the volunteers’ functional motives to the activities that they are asked to perform |
| Gift exchange and high performance Human Resource Management theory [21]  Private benefits model, investment model [22] | Retention and performance will be higher and turnover lower in organisations with comparatively high investment in, and care for, employees | Provide bundles of incentives, training skills  Invest in support |
| Social exchange theory [23] | HRM practices influence the employees’ perceptions of organisational support, which in turn induce positive work attitudes and behaviours based on the norm of reciprocity | Allow practices such as career development opportunities, investment in training, etc. that signal the organisation’s commitment to their volunteers |
| Equity theory [24] | Employees expect equity between what they give to the organisation and what they receive in return  If employees feel that their inputs such as efforts, commitment, loyalty, trust, and enthusiasm for the organisation are fairly rewarded by financial and non-financial benefits or incentives, they remain motivated and continue to provide inputs towards higher productivity. Employees get de-motivated due to the perceived absence of such equity | Provide bundles of incentives, training skills  Invest in support |
| Consistency theory [25] | Practices need to be consistent for high retention and performance | Guarantee internal consistency among different HRM practices  Guarantee contextual consistency between HRM practices and organisational context |
| Organisational commitment [26] | Organisational commitment contributes to better job-related attitudes, higher job satisfaction and better organisational performance.  The type of organisational commitment that encompasses accepting the organisational goals, being committed to the organisation, and feeling engaged with and attached to the organisation appears to be facilitated by autonomous motivation (link with SDT theories) | Develop institute practices that facilitate positive psychological links between organisational and employee goals |
| Life span theory and life course theory [27] | .  Volunteering is associated with life course factors, and changes in these factors are related to participating, and to starting and stopping volunteering  Different factors affect volunteering at different life stages, so some factors may be particularly important in the middle adult years, and other factors which may be more important in later adulthood and in retirement  Volunteering can help teenagers develop positive self-identities  By volunteering, people can feel useful to the society and gain an overall sense of integrity about their lives. | Recruitment: allow enough time to listen to volunteers  Appropriate incentives: provide appropriate incentives according to the life stage of the volunteers |
| The three stage of volunteer duration [28]  The volunteer stages and transition model [29] | Different factors explain volunteer retention and attitude depending on how long a volunteer has been with the organisation  Satisfaction of initial motives is a better predictor of retention in the short term, one’s commitment to the organisation and how much one identifies with it predict long term retention | Foster-managerial practices that cater to the immediate expectations of a volunteer in the short term, while ensuring the development of organisational commitment in the medium term, and the development of role identity in the long term |

References

1. Turner JC, Reynolds KJ. The story of social identity. In: Postmes T, Branscombe NR, editors. Rediscovering social identity: core sources. UK: Psychology Press; 2010.
2. Strachan DL,  [Källander K](http://www.ncbi.nlm.nih.gov/pubmed/?term=K%C3%A4llander%20K%5BAuthor%5D&cauthor=true&cauthor_uid=25925007), Nakirunda M, Ndima S, Muiambo A, Hill Z, et al. Using theory and formative research to design interventions to improve community health worker motivation, retention and performance in Mozambique and Uganda. Hum Resour Health. 2015;13:25.
3. Fawcett SB, Paine-Andrews A, Francisco VT, Schultz JA, Richter KP, Lewis RK, et al. Using empowerment theory in collaborative partnerships for community health and development. Am J Community Psychol. 1995;23(5):677-97.
4. Freire P. Pedagogy of the oppressed (M. B. Ramos Trans.). New York: Continuum;1970.
5. Freire P. Education for critical consciousness. New York: Continuum International Publishing Group; 2005.
6. Bandura A. Social learning theory. Englewood Cliffs, NJ: Prentice Hall; 1977.
7. van der Bijl JJ, Shortridge-Baggett LM. The theory and measurement of the self-efficacy construct. Res Theory Nurs Pract. 2001;15:189–207.
8. Rowe AK, de Savigny D, Lanata CF, Victoria CG. How can we achieve and maintain high-quality performance of health workers in low-resource settings? Lancet*.* 2005;366:1026–35.
9. van der Bijl JJ, Shortridge-Baggett LM. The theory and measurement of the self-efficacy construct. Res Theory Nurs Pract. 2001;15:189–207.
10. Rycroft-Malone J, Fontenla M, Bick D, Seers K. A realistic evaluation: the case of protocol-based care. Implement Sci. 2010, 5:38.
11. Hasenfeld, Yeheskel, Rino Patti. "The Utilization of Research in Administrative Practice." New York: Hawworth Press; 1992:221-39.
12. Frey BS, Jegen R. Motivation crowding theory: a survey of empirical evidence. J Econ Surv. 2001;15:589–611.
13. White RW. Motivation reconsidered: the concept of competence. Psychol Rev. 1959;66:297–333.
14. Unger LS. Altruism as a motivation to volunteer. J Econ Psycol. 1991;12(1):71-100.
15. Ziemek S. Economic analysis of volunteers’ motivation-A cross-country study. J Socio Econ. 2006;35(3):532-55.
16. Andreoni, James. Impure altruism and donations to public goods: A theory of warm-glow giving. Econ J. 1990;100:464-77.
17. Schiff J. Charitable Giving and Government Policy: An Economic Analysis. New York: Greenwood Press;1990.
18. Sherr ME. Volunteerism and Human Behaviour Theory. In: Social work with volunteers. <http://lyceumbooks.com/pdf/sw_w_volunteers_chapter_03.pdf>. Accessed 31 March 2016.
19. Deci EL, Ryan RM. Intrinsic motivation and self-determination in human behaviour. New York: plenum; 1985.
20. Clary EG, Snyder M, Ridge R, Stukas AA, Copeland J, Haugen J, et al. Understanding and assessing the motivations of volunteers: a functional approach. J Pers Soc Psychol. 1998;74:1516–30.
21. Cheal DJ. The gift economy. New York: Routledge; 1988:1–19.
22. Ziemek S. Economic analysis of volunteers’ motivation-A cross-country study. J Socio Econ. 2006;35(3):532-55.
23. Emerson R. Social exchange theory. Annu Rev Sociol. 1976;2:335–62.
24. Adams JS. Inequity in Social Exchange. In: Advances in Experimental Social Psychology. New York: Academic Press;1965.
25. Festinger L. A theory of cognitive dissonance. Stanford, CA: Stanford University Press; 1957.
26. Meyer JP, Allen NJ. A three-component conceptualization of organizational commitment. Hum Resour Manag Rev 1991;1:61–89.
27. Sherr ME. Volunteerism and Human Behaviour Theory. In: Social work with volunteers. <http://lyceumbooks.com/pdf/sw_w_volunteers_chapter_03.pdf>. Accessed 31 March 2016.
28. Chacon F, Vecina ML, Davila MC. The three stage model of volunteers’ duration of service. Soc Behav Pers. 2007;35(5):627-42
29. Haski-Leventhal D, Bargal D. The volunteer stages and transitions model: organisational socialisation of volunteers. Human Relations. 2008;61(1):67-102.
