# Supplementary material for: Understanding the performance of community health volunteers involved in the delivery of health programmes in underserved areas: a realist synthesis
Source: Implement Sci. 2017 Feb 16;12:22. doi: 10.1186/s13012-017-0554-3 (PMC5314678; doi:10.1186/s13012-017-0554-3)
Supplement: Additional file 2: — Overview of the methodology used, contextual factors identified, the interventions, the mechanisms triggered and the outcomes in the selected studies. (PDF 502 kb) [file 13012_2017_554_MOESM2_ESM.pdf]

Additional File 2: Overview of the methodology used, contextual factors identified, the interventions, the mechanisms triggered and the outcomes in the selected studies

Acronyms and abbreviations

ACT	Artemisinin combination therapies
ART	Antiretroviral therapy
ASW	Adherence support workers (Zambia)
CBD	Community-based distribution
CBO	Community-based organisation
CBPR	Community-based participatory research
CBSV	Community-based surveillance volunteer (Ghana)
CHA	Community health advisor (San Diego)
CHV	Community health volunteer (Kenya)
CHW	Community health worker
CVW	Community volunteer worker (Uganda)
ETS	Environmental tobacco smoke
FCHV	Female community health volunteer
HMM	Home-based management of malaria
LHW	Lay health worker(Zimbabwe)
MCA	Malaria control assistant (Sudan)
MNCH	Maternal, neonatal and child health
NGO	Non-governmental organisation
PHC	Primary Health Care
PHW	Peer health worker (Uganda)
PLWHA	People living with HIV/AIDS
RDT	Rapid diagnostic test
VDC	Village Development Committee (Nepal)

1. Carter-Pokras OD, Jaschek G, Martinez IL, Brown PB, Mora SE, Newton N, et al. Perspectives on Latino lay health promoter programs: Maryland, 2009. I. Am J Public Health. 2011.

Setting

Latin America, Maryland

Context

- Poor beneficiary population
- Underserved area: cultural and financial barriers to care
- High level of interest and respect generated by promoters’ programmes
- Personal growth and status are highly valued

Intervention characteristics

- Lay health promoters: volunteer health promoters within community-based organisations
- Intervention by peers who are socio-culturally competent
- Development of an advisory board from within the target population
- Selection of promoters who are highly motivated, have good communication skills, are enthusiastic and express desire to help the community
- Careful selection to ensure that promoters are able and willing to fulfil their role
- Training that is skills-based, using interactive educational methods and curriculum that includes specific health and personal development skills
- Financial incentives (stipends, cell phone, babysitting money) and non-financial (recognition, opportunity for personal growth, additional skills)
- Participation costs the promoters time and money

Mechanisms

- Skills-based training using interactive methods and promoters’ experience enhances self-efficacy and self esteem
- Monetary incentives trigger high level of accountability towards the programme
- Non-monetary incentives are also highly valued and may have motivated CHVs

Outcomes

- Programme positively affected the lives of health promoters
- Lay health promoters are satisfied
- Promoters have good competences: provide accurate information and educate the community
- Programme is of quality and consistent

Conclusion by the authors

Needs assessments, identification of appropriate promoters and a significant amount of training contributed to a successful health promoter programme where lay promoters are able to communicate health information in a competent way.

Methodology

- Qualitative

Sample

- 10 lay health promoters
- Programme coordinators

2. Daoud N, Shtarkshall R, Laufer N, Verbov G, Bar-El H, Abu-Gosh N, et al. What do women gain from volunteering? The experience of lay Arab and Jewish women volunteers in the Women for Women's Health programme in Israel. Health Soc Care Community. 2010.

Setting

Israel: Jewish and Arab communities

Context

- Poor beneficiary population with poor health practices and outcomes
- These two ethnic groups (Jewish and Arab) live separately and have few opportunities to meet on an equal footing
- Volunteering by Arab women is unacceptable
- Arab women's traditional role as health caregiver is valued
- Arab women are considered marginalised ethnic minority in Israeli society and have few life opportunities in both the personal and public domain
- Jewish women volunteers have more life experience, are older and have more leisure time
- Jewish women volunteers may have job responsibilities outside home and thus less time to deal with themselves and quality of life of their families

Intervention characteristics

- Lay volunteer women mobilise health activities in their communities
- Culturally appropriate intervention where women act as role models
- Training skills-based
- Participatory approach
- Group cohesion and collaboration towards a positive common goal: the possibility for the lay volunteers to share their experience and to solve problems with other volunteers from the other ethnic group
- The time spent volunteering did not detract from women's effectiveness as homemakers

Mechanisms

- The design of the intervention triggers an increase in self-esteem, a feeling of self-worth and a feeling of being respected by the community
- Training and participation in the activities of the programme (offering new social opportunities) enhance self-confidence
- Arab women can fill a psychological need for self-satisfaction (volunteering work did not take away time from the family, but rather added to its positive benefit)
- Jewish women fulfil advisory roles to other women in the community and replace the empty nest with volunteering
- The possibility of collaborating increases the sense of belonging within the community

Outcomes

- Volunteers have better competences (improvement in knowledge and skills, can communicate accurately, are able to navigate the health care system assertively)
- Volunteers are satisfied with the programme
- The number of volunteers that participate in the programme increased

Conclusion

Volunteering in community-based health promotion programmes can be an empowering experience for lay women with lower previous social standing, providing them with opportunities for greater social participation and a wide range of benefits. Participatory approaches can intensify the project's appropriateness to the cultural context.

Methodology

- Qualitative

Sample

- 45 lay volunteer women

3. Alfaro-Trujillo B, Valles-Medina AM, Vargas-Ojeda AC. Profiles, perceptions and motivations of Community Health Workers of NGOs in a border city of US-Mexico. J Community Health. 2012 Jun;37(3):583-90.

**Setting**<br>Border city of US-Mexico
**Context**<br>• Community interest in public welfare (the concept of health is assumed to be an offer of medical services, to which the community responds well and gives support)<br>• Poor beneficiary population with high level of migrants with no legal status and high morbidity<br>• Low level of education<br>• Weak health system<br>• Community health worker (CHW) initial level of commitment to achieving community participation
**Intervention characteristics**<br>• CHW cultural competences<br>• CHW tasks with polyvalent focus: distribution of informational materials and promotion of health fairs<br>• Continuing education and supervision with educational workshops<br>• Permanent recognition of CHW work (educational offering, certificate, special event, verbal appreciation)<br>• CHWs are treated as employees: employee status is clear for CHW<br>• Economic compensation covers transportation costs to the communities in which the health interventions are conducted<br>• In kind compensation
**Mechanisms**<br>• The perception of the effects of the intervention (early and visible results) triggers self-efficacy<br>• The intervention that offers free services is supported by the community, triggering perception of community support and recognition<br>• The on-job training with certificate triggers changes in the self-esteem/life plan and a better understanding of community need<br>• The non-governmental organisation (NGO) institutional relationship triggers a sense of belonging to the organisation (CHWs feel like formal workers) and the perception of equal institutional relationship among workers triggers a feeling of self-worth, self-efficacy and a sense of higher social recognition<br>• CHWs perceive the economic compensation as insufficient for the services rendered and they search for other sources of income to fulfil their needs. However, for others, the monetary factor adds motivation toward community participation
**Outcomes/conclusion**<br>• The NGO's recognition for the CHWs' work contributes to CHW work satisfaction, attachment and a long-term commitment with the organisation<br>• Continuing education is the most prized element for continuity in community work<br>• The most unsatisfying aspect of work for CHWs is the system of benefits and compensation and this contributes to the reduction of their involvement
**Methodology**<br>• Mixed (qualitative focus group discussion, quantitative surveys)<br>• Cross sectional study design
**Sample**<br>• 121 CHWs for the structured questionnaire and 18 CHWs for the focus groups

4. Campbell C, Gibbs A, Maimane S, Nair Y. Hearing community voices: grassroots perceptions of an intervention to support health volunteers in South Africa. SAHARA J. 2008.

Setting
South Africa

Context
- Rural setting
- Poor beneficiaries with high AIDS-related illness and death in the community
- Geographical/financial and cultural barrier to care (traditional beliefs about illness causation and ignorance on HIV/AIDS)
- Level of HIV stigma is high and community leaders show little interest in HIV/AIDS in their community
- Weak health system, bad access to referral services and scarcity of health workers
- The municipality and local health department play a minimal role in this particular community and residents are effectively under the control of a traditional chief and counsellor
- Limited role of external agencies

Intervention characteristics
- Most volunteers are women who had already been offering home nursing support to households affected by AIDS
- Home nursing care
- Intervention uses democratic principles (the hosting organisation does not interfere in the internal management of the volunteer team), ensuring involvement by a wide range of community members, volunteers' active involvement in project planning, implementation and evaluation and ensuring that the volunteers are active participants in supportive social networks
- The training includes workshop facilitation (skills-based training about HIV/AIDS treatment, care and support; management and leadership in general)
- A number of public events developed, including graduation ceremonies for health volunteers on completion of training
- The programme provides basic uniform/small temporary stipends
- Link between volunteers and local leaders strengthened through training of a wide range of local groups
- Link with external agencies strengthened through partnership meetings and development of a coordinating body

<u>Context during project implementation: lack of ownership and involvement in the project among community members</u>
- Lack of support from local leaders and limited role played by external agencies in supporting the project (no understanding of the need for more collective community-level responses, the male leaders of the health volunteers had not developed new and more gender-neutral ways of sharing power and decision making in the team)
- AIDS-related stigma still perceived as a major barrier and people in the community express an inability to deal with HIV/AIDS because of the scale of epidemic in their country
- Still limited support from external agencies

Mechanisms
- Training and participation in the project trigger volunteer self-confidence and self-efficacy
- Since being trained, volunteers were highly respected in the community. The feeling of recognition from the community also triggers volunteers' self-confidence.
- Basic uniform and graduation ceremonies contribute to community recognition and trigger self-esteem/sense of pride
- Sense of pride in the work because of stipends
- The context during project implementation triggers less sense of ownership by female volunteers
- Volunteers' lack of awareness about the partnership with external agencies.

Outcomes
- Volunteers' job related satisfaction
- Good competences (knowledge about HIV/AIDS, communication skills)
- Increased access to government projects
- No volunteer participation in every aspect of project functioning and no ability to exercise leadership in areas beyond HIV/AIDS

Conclusion
The project contributed to running a more effective home nursing service (success in the delivery of home nursing) but the efforts would not be sustainable in the long term due to lack of support for volunteers both within and outside the community (failure to prepare female volunteers to sustain the activities once the formal life of the project had ended).

Methodology
Qualitative

Sample
34 volunteer health workers

5. Woodruff SI, Candelaria JI, Elder JP. Recruitment, training outcomes, retention, and performance of community health advisors in two tobacco control interventions for Latinos. J Community Health. 2010;35:124-34.

**Setting**<br>San Diego
**Context**<br>• Poor beneficiary population<br>• High disease burden related to smoking behaviour and environmental tobacco smoke (ETS) exposure (passive smoking)<br>• Low level of education<br>• Barrier associated with health care access and culturally appropriate smoking cessation treatment (minority groups tend to rely on informal sources for health information)<br>• Community health advisors (CHAs) have a strong sense of community. Usually female, generally in their early forties, married, born and educated in Mexico, of relatively low acculturation level, had modest incomes, and had prior community experience
**Intervention characteristics**<br>• Culturally sensitive tobacco control interventions by indigenous CHAs<br>• Children focus: designed to reduce exposure to ETS among low-income Latino children<br>• Intervention home-based and accessible<br>• Training over a one-month period with flexibility in timing and content. It includes didactic methods, role-playing, skills development, problem solving approach, motivational interviewing techniques, and ongoing mastery testing of the intervention curriculum (the curriculum focuses on positive reinforcement, stimulus control, modelling, social support, problem solving, and practical skills and techniques for quitting smoking)<br>• Ensure safety and build camaraderie<br>• Moral and technical support: meetings and feedback, build capacity in areas of interest to them for future work<br>• Incentives: modest stipends, awards, celebration, recognition ceremonies, current contact information collected from CHAs for referral to other active projects
**Mechanisms**<br>• The training, participation in the intervention and the perception of positive visible results of the intervention trigger self-esteem and self-efficacy<br>• Realisation of being valuable to one's community<br>• The hope for employment and the incentives may have motivated CHAs to perform better<br>• Assurance that there is a system of back-up in time of need and perception of sufficient training
**Outcomes/conclusion**<br>• CHA level of efforts and professionalism increase<br>• Good knowledge and attitudes related to the programme to be delivered<br>• Good overall performance in achieving the goals of the project<br>• Good retention and satisfaction
**Methodology**<br>• Randomised controlled trial
**Sample**<br>• 35 CHAs

6. Takasugi T, Lee ACK. Why do community health workers volunteer? A qualitative study in Kenya. Public Health. 2012; 126(10):839-45.

<table>
<tr><td colspan="2">Setting
Kenya</td></tr>
<tr><td colspan="2">Context
Rural setting
High mortality and morbidity rate
Low levels of education and health knowledge in the population
CHW intervention is valued
CHWs are drawn from relatively poor socio-economic groups. They are doubly burdened by poverty and having to carry out unpaid work that can be role consuming
The government scheme and the scheme of NGOs that support CHWs may lead to employment</td></tr>
<tr><td colspan="2">Intervention characteristics
Home-based care, such as first aid and the prevention and management of minor ailments
Items are provided to CHWs as part of their work, such as badges and shirts
Lack of resources and transportation, no monetary incentives</td></tr>
<tr><td colspan="2">Mechanisms
CHWs feel they gain respect from the community for this valued role, which also contributes to CHWs' sense of self-pride
The provision of badges and shirts enhances a feeling of being identified as CHWs by the community
Training triggers self-confidence in their skills and knowledge
CHVs agreed that monetary incentives would undoubtedly improve their motivation to work
The hope that the voluntary role would lead to salaried employment in time motivates CHWs to perform better
CHWs felt that the lack of resources to perform their work and lack of transportation undermined their role as it meant they could not live up to the expectations of the communities
The loss of trust by the community in their services decreases CHWs' level of motivation</td></tr>
<tr><td>Outcomes
Monetary and non-monetary incentives (personal growth, community recognition) influence the motivation to perform well and the retention of CHWs</td><td>Conclusion
This study indicates that the maintenance of a well-motivated and effective community health workforce requires consideration of some form of reward.</td></tr>
<tr><td colspan="2">Methodology
Qualitative study using 6 focus group discussions</td></tr>
<tr><td colspan="2">Sample
23 CHWs</td></tr>
</table>

7. Glenton C, Scheel I, Pradhan S, Lewin C, Hodgins S. The female community health volunteer programme in Nepal: Decision makers' perceptions of volunteerism, payment and other incentives. Soc Sci and Med. 2010; 70(12):1920-7.

Setting

Nepal

Context

- Rural setting
- High level of disease and rate of mortality
- Tradition of volunteering as moral behaviour
- Lack of respect for paid government workers
- Lack of knowledge on health
- Low level of education and few job-related opportunities
- Female CHVs (FCHW) willing to work in their own time and about 5 hours a week

Intervention characteristics

- Community embedment: volunteers are selected by, and work within, the community
- 18 days of initial training as well as refresher training
- Incentives provided by the government including transport stipends for training, access to micro-credit funds, ID card and celebration events
- Village Development Committees (VDCs) may choose to give incentives such as umbrellas and bicycles to local FCHVs while non-governmental organisations also provide incentives, including direct payment for specific activities

Mechanisms

- Participation in the intervention enhances a sense of personal satisfaction, a sense of improvement in social status and a sense of social mindedness

More specifically:

- Benefiting from visible symbols enhances a feeling of social recognition
- Rewards that are distinguishable from a government salary and social respect trigger a feeling of being acknowledged for their contribution
- Training and refresher training trigger a sense of self-efficacy

Outcomes

- Low attrition rate
- Improvement in community capacity to claim rights

Conclusion

A high level of attention should be paid to ensuring that the expectations of CHWs, programme managers and policy makers are in alignment if low attrition and high performance are to be achieved (effectiveness and sustainability of the programme)

Methodology

- Qualitative

Sample

- 6 FCHVs and 19 non-volunteer stakeholders, including policy-makers and programme managers

8. Olang'o CO, Nyamongo IK, Aagaard-Hansen J. Staff attrition among community health workers in home-based care programmes for people living with HIV and AIDS in western Kenya. Health Policy. 2010;97:232–7. doi: 10.1016/j.healthpol.2010.05.004.

Setting

Kenya

Context

- Poor beneficiary population
- HIV burden
- Geographical barrier to care
- Lack of human resources at health facilities
- Young population: competing demand in the market
- Men are expected to be the primary providers for the family (need money to support their family, no spare time for visiting patients in the village)
- Gender-power differences favour men, lack of spousal support
- Intra-house gender power relation: care services are regarded as being in the female domain
- Most community-based organisations (CBOs) are welfare-based and financially unstable

Intervention characteristics

- Home-based care
- The NGOs do not provide adequate programmatic and managerial support based on fairness and equity to CHWs (power relations between the officials and CHWs)
- Lack of clear selection criteria

Process of implementation

- Patients and their relatives expected the CHWs to share their food and other material goods as well as money to facilitate transport of patients to health facilities
- The NGO could not help out the CHWs because of their poor financial base

Mechanisms

- Men feeling inability to discharge their responsibility (in term of being income providers)
- Lack of transparency among the NGO staff: the CHWs feel that access to improve skills is denied and opportunities are selectively dished out
- Younger CHWs are constantly looking for opportunities to improve their work
- Feeling of lack of spousal support for women CHWs
- In the cultural context, where care services are regarded as being in the female domain, the involvement of male CHWs to provide home-based care triggers a feeling of lack of recognition from the community
- CHWs who cannot meet community expectations trigger a feeling of lack of recognition from the community

**Outcomes**	**Conclusion**
• High attrition rate	The reasons for dropout included: the cultural environment within which CHWs operated; lack of adequate support from area NGOs; poor selection criteria for CHWs; and power differences between NGO officials and CHWs which fostered lack of transparency in the NGO's operations.

Methodology

- Qualitative (ethnographic data collected through participant observation, focus group discussions and in-depth interviews)

Sample

- 100 with 30 CHWs, NGO staff and health care providers and 70 people living with HIV/AIDs (PLWHA)

9. Dil Y, Strachan D, Cairncross S, Korkor AS, Hill Z. Motivations and challenges of community-based surveillance volunteers in the northern region of Ghana. J Community Health. 2012;37(6):1192-8. doi: 10.1007/s10900-012-9569-5.

Setting

Ghana, northern region

Context

- Community identify health to be the most important aspect in human life
- Resource-limited settings
- Community-based surveillance volunteers (CBSVs) form part of Ghana's Community Based Health and Planning Services strategy
- CBSV background: either farmers or teachers and tend to be older members of the community; understand that their role is voluntary

Intervention characteristics

- Community selection of the volunteers
- One-off payments for activities such as participating in national immunisation days and for attending training meetings
- Provision of equipment such as raincoats, identifiers such as tee-shirts, certificate or token of appreciation (with material value, something that they can be proud of)
- Training sessions and workshops
- Supportive supervision from formal health staff

Process of implementation

- Lack of community cooperation during mobilisation in some areas and often insufficient understanding of the role of CBSVs by the community
- Problems with transportation and equipment
- Lack of supervision during the rainy season
- Lack of payment for ad hoc tasks, lack of adequate compensation when expected (delays and lack of payment can result from health workers taking some of the money)
- Difficulties conducting both volunteer and farm work

Mechanisms

- Participation in the programme, being able to provide health information and visible results triggers sense of duty, active desire to help their families and communities, feeling of respect from the community and a feeling of pride
- Training and actively seeking the health and education of the community also trigger a sense of self-efficacy
- Being selected by the community influenced the sense of duty felt by CBSVs as well as the pride they felt for the role and their motivation
- The recognition/support and incentives (small tokens) from the organisation trigger a feeling of being part of the formal health system, and a sense of being valued by the local government
- Incentives with material value are appreciated and are a strong motivating factor, especially as CBSVs would often get behind on their farm work due to their duties

Difficulties during process of implementation

- Not receiving promised incentives was demoralising and distressing for some and may lead to poor performance and commitment
- Expectations including provision of equipment to aid the CBSVs in their duties were not met and this lowered the morale of volunteers

Outcomes/conclusion

- Importance of promoting a strong relationship with the community for better retention and quality of care to be achieved
- Volunteers must have a clear picture of what to expect from the role, and that these expectations are met to ensure morale and performance remains high
- Importance of providing small tokens to show the volunteers that they are valued

Methodology

Qualitative (semi-structured interviews)

Sample

28 CBSVs, 33 health service staff

10. Alam K, Tasneem S, Oliveiras E. Performance of female volunteer community health workers in Dhaka urban slums. Soc Sci Med. 2012;75(3):511-5.

Setting

Dhaka, Bangladesh

Context

- Poor beneficiary population
- Health workforce crisis
- Urban slums setting with unstable urban slum dwellers
- CHWs were poor and it was difficult to support their families (minimum of income important to address their daily needs)

Intervention characteristics

- BRAC (NGO) involves CHWs
- Provision of financial incentives that increase CHW income (an increased allowance for attending refresher training; a one-time incentive package for pregnancy identification, delivery and new-born care; and supply of drugs and commodities at lower cost)

Process of implementation

- Positive community feedback /support and cooperation from their family

Mechanisms

- Involvement in the programme enhances social prestige (CHWs receive more greetings, more honour, more participation in community decision making and more invitations). Therefore, the CHWs felt that the community members knew them, respected them and looked to them to address their health needs
- CHWs feel that the money they earn as CHWs is important to their families' maintenance

Note: the feeling of social prestige can be challenged in this urban area where CHWs worked among comparatively unstable urban slum dwellers

Outcomes

- Active participation in the core activities of the programme

Conclusion

Financial incentives were the main factor linked to the activity of CHWs. In addition, social prestige and positive community feedback to the CHWs were important non-financial factors associated with level of activity.

Methodology

- Mixed (interviews and quantitative survey)
- Descriptive correlational design

Sample

542 CHWs

11. Alam K, Tasneem S, Oliveras E. Retention of female volunteer community health workers in Dhaka urban slums: a case-control study. Health Policy Plan. 2012;27(6):477-86.

<table>
<tr><td colspan="2">Setting
Dhaka, Bangladesh</td></tr>
<tr><td colspan="2">Context
Poor beneficiary population
Health workforce crisis
Urban slums setting
Local labour market in urban areas: competing environment
CHW were poor and it was difficult to support their families (minimum of income important to address their daily needs),
CHWs expectations (or not) of income and CHW level of household responsibilities are important factors
The concept of volunteers being used by BRAC is not institutionalised in line with the standard volunteer definition: for instance, no guidelines in terms of the expected duration of the participation</td></tr>
<tr><td colspan="2">Intervention characteristics
BRAC (NGO) involves CHWs
Provision of financial incentives (an increased allowance for attending refresher training; a one-time incentive package for pregnancy identification, delivery and new-born care; and supply of drugs and commodities at lower cost)
Development of networking with key social institution
<u>Process of implementation</u>
Positive community feedback/support and cooperation from their family
Communication gap between BRAC staff and volunteers and different expectations from the programme and CHWs</td></tr>
<tr><td colspan="2">Mechanisms
CHWs feel that the money they earn as CHWs is important
Involvement in the programme enhances an increase in social prestige (CHWs receive more greetings, more honour, more participation in community decision making and more invitations). Therefore, the CHWs felt that the community members knew them, respected them and looked to them to address their health needs
Communication gap between BRAC staff and volunteers led to false expectation among some volunteer CHWs that their role was a paid job rather than a voluntary role (may lead to high dropout)</td></tr>
<tr><td>Outcomes</td><td>Conclusion
Financial incentives were the main factor linked to CHW retention (CHWs who joined with the expectation of income were almost twice as likely to remain.) Social prestige, community approval and household responsibilities were important factors associated with CHW retention.</td></tr>
<tr><td colspan="2">Methodology
Mixed (group discussions and quantitative survey
Case control design</td></tr>
<tr><td colspan="2">Sample
688 CHWs</td></tr>
</table>

12. <u>Jack BA, Kirton JA, Birakurataki J, Merriman A. The personal value of being a palliative care Community Volunteer Worker in Uganda: a qualitative study. Palliat Med. 2012;26(5):753-9. doi: 10.1177/0269216311413628.</u>

Jack BA, Kirton JA, Birakurataki J, Merriman A. A bridge to the hospice: The impact of a Community Volunteer Programme in Uganda. Palliat Med. 2011;25:706-15. doi:10.1177/0269216310397566.

**Setting**<br>Uganda
**Context**<br>• Rural setting<br>• Cultural wish to help people<br>• Community members had prior personal experience with diseases or palliative care: volunteers had experienced multiple losses, particularly with the HIV/AIDS death rates in the previous decade<br>• Health and social needs<br>• Lack of access to education<br>• Volunteers' background: young, majority have not undergone further or higher education, more males
**Intervention characteristics**<br>• The community volunteer workers (CVWs) are selected by senior members of the local community and meet the following criteria: completed a basic education, identified as being respected and trustworthy, and available to volunteer for about 6 hours a week<br>• Training: 6-day non-residential course that covers an overview of the fundamentals of palliative care, practical aspects of home nursing care and communication skills. Ongoing education and support are provided by the hospice team, with monthly meetings being held, and contact via mobile phone for day-to-day support and advice<br>• The volunteers are provided with a bicycle and maintenance fund sundries (a kit that contains basic practical items such as antiseptic, gloves, etc.) and a uniform tee-shirt (badge of office) denoting their volunteer work with Hospice Africa Uganda.<br><br><u>Process of implementation</u><br>• A partnership between the CVWs and the hospice team
**Mechanisms**<br>• Participating in the programme and receiving recognition signs trigger high level of personal pride, improvement in social status and feeling of community respect
**Outcomes/conclusion**<br>• Good quality of services, improvement in access to health care, good retention
**Methodology**<br>• Qualitative (group discussion and individual interviews)
**Sample**<br>32 CVWs and 11 hospice clinical teams

13. Ochieng BM, Kaseje DO, Mala SJ, Mumbo HM, Aila FO, Odera O. Motivational drivers for non-skilled Kenyan community health volunteers. Int J Asian Socl Sci.2012; 2(9):1477-83.

Setting
Kenya

Context
- Religious values
- Belief that humanity requires dignified living
- Health inequities in access to health services (poor infrastructure, inadequate health workforce, inaccessible products, etc.)
- Bad health outcomes with high mortality rate
- Inability of education system to equip school leavers with sufficient skills for employment
- CHVs background that matters: age, and gender role (availability of time, role an individual is expected to assume, type of services CHVs offer), existing leadership of the CHVs within the community, CHVs' past unpleasant experiences

Intervention characteristics
- Access to training opportunities by volunteers for acquisition of knowledge in relation to the specific tasks performed by CHVs
- Supervision and follow-up support: effective facilitation process for volunteerism

Mechanisms
- Volunteering triggers a feeling of fulfilling religious values and serving humanity
- The desire to give service to humanity and to witness changes in the well-being of the people they serve over the years contributes to their retention
- Motivation also comes from the nature of benefits that the immediate families of the CHVs are likely to attain
- Visible results enhance recognition of the CHVs by the community
- CHVs discovered through the process of volunteerism that they had inborn leadership qualities that enable them to coordinate and influence others to change, or more generally to feel confident in their ability to perform the task

Outcomes/conclusion
- Active in offering volunteering services and good retention

Methodology
- Mix (quantitative survey and qualitative in-depth interviews and focus group discussions)
- Cross-sectional and descriptive study

Sample
261 CHVs

More specific CMOs from this paper according to age and gender issues:

**Context: age and gender issues**	**Intervention characteristics and context of implementation**	**Mechanisms**	**Outcomes**
Volunteerism is valued by women as a critical part of a woman's responsibility to society	Volunteerism	Building social capital	Active in offering volunteering services
Women look after their homes	Voluntary work: few hours in a day	Availability of time	Active in offering volunteering services
Men younger, are the breadwinners in most household's: spend more time engaged in paid work	Unpaid work	No time availability	Not active in offering volunteering services
Older men have settled in their rural homes and have experience of CHVs' activities and leadership<br>Valued by the community for provision of food security, education	Volunteerism<br>Type of service CHVs offer: food security, education	Availability of time/community recognition	Active in offering volunteering services
Women valued for maternal, neonatal and child health (MNCH), HIV, malaria services	Type of service CHVs offer: MNCH, HIV/AIDS, malaria	Community recognition	Active in offering volunteering services

14. Yakam JC, Gruénais ME. Involving new actors to achieve ART scaling-up: difficulties in an HIV/AIDS counselling and testing centre in Cameroon. Int Nurs Rev. 2009;56:50-7.

Setting
Cameroon

- Urban setting (far north province of Cameroon)
- High HIV/AIDS-related mortality (HIV/AIDS is a disease that disrupts lives, and although antiretrovirals have become more affordable there is still very low antiretroviral therapy (ART) coverage and high shortage of health workers)
- Poor beneficiary population and few employment opportunities
- CHWs do not have any specific qualifications apart from the fact that they all are PLWHA and so have an 'incorporated' expertise. Most are women (only one man is part of the group); some are widows whose husband died from HIV/AIDS. Before their involvement in this activity, they were homemakers

Health system in Cameroon

- Weak, dysfunctional and unregulated health system (human resources for health crisis); informal payments and poor provider attitudes are common: the difficult working conditions of health personnel, specifically nurses, make models of ART delivery highly doctor-intensive. Health workers (nurses) are paid salaries well below subsistence levels.
- Absence of specific role definitions and precise procedure for task shifting
- No accreditations of training

Intervention characteristics

- CHWs are selected community members who are trained in general primary care functions or in specific activities
- Peer model intervention
- Activities are based in HIV and testing counselling centres
- Opportunities for per diem for seminar or training

Process implementation
Relationships between CHWs and other health workers are ill defined. CHWs are often poorly supported in terms of follow-up training and regular supervision (nurses see CHWs as a threat).

Mechanisms

- A sense of relatedness with and accountability to the beneficiaries contributes to retention
- CHWs' position and involvement in the intervention helps them discover their 'hidden' skills and value. This triggers a feeling of self-efficacy and self-worth
- Minimum fees or incentives that have financial value are considered important

Difficult relation between CHWs and nurses: nurses seeing CHW as a threat

- CHWs struggle over definition and identity: difficulty for CHW to prove their usefulness and their legitimacy
- Creates tension, frustration and problems related to nurses' position within the existing hierarchies. CHWs do not have trusting relationship with formal staff (nurses)

**Outcomes**	**Conclusion**
• The good link with community members can contribute to job related satisfaction and retention	Involving new actors, such as CHWs, in an unregulated health system could lead to their settling in and the exclusion of formal health workers. It could create tension, frustration, and problems related to legitimacy and position of these new actors within the existing hierarchies. Poor relationships and the lack of supervision contribute to poor performance and dissatisfaction (bad health practices).

Methodology
Qualitative interviews, direct observation with key informants (interactions between co-workers, patients and health workers)

Sample
Unknown

15. Vissman AT, Eng E, Aronson RE, Bloom FR, Leichliter JS, et al. What do men who serve as lay health advisers really do?: Immigrant Latino men share their experiences as Navegantes to prevent HIV. AIDS Educ Prev. 2009;21:220-32.

<table>
<tr><td colspan="2">Setting
Central North Carolina</td></tr>
<tr><td colspan="2">• Low use of preventive services
• Poor health status
• Cultural and financial barriers to accessing counselling and testing (price and negative expectations as common barriers to condom purchase)

<u>Target of the intervention:</u>
• Male immigrant Latino communities (18—44 years of age) belonging to the soccer league
• High case rate of AIDS, HIV, gonorrhoea and chlamydia
• Low level of education
• The current capacity of public health departments and other health care providers is limited because of no prior history of planning for and providing services that are bilingual and bicultural</td></tr>
<tr><td colspan="2">Intervention characteristics
• Culturally appropriate intervention with peer to peer model (navegantes distribute culturally appropriate materials, correct misconceptions, using existing networks and informal communication styles, building community member skills, and serving as role models)
• Problem-solving training oriented to public-speaking skills with certificate upon graduation
• Supported by a community-based participatory research (CBPR) partnership that included a project coordinator (native Spanish speaker with a similar immigration history)
• Navegantes received materials (e.g. condoms and other risk reduction materials) and held monthly meetings to plan, coordinate, and evaluate their activities</td></tr>
<tr><td colspan="2">Mechanisms
• Increase of self-recognition as opinion leaders
• The training and the certificate trigger a feeling of self-worth
• Training and participation in the programme, plus the use of culturally appropriate materials enhance a sense of self efficacy/self-confidence and capacity to engage their peers in sexual health discussion. It also triggers a feeling of being perceived as competent
• Sense of autonomy</td></tr>
<tr><td>Outcomes
• CHW increased knowledge and skills in HIV and sexually-transmitted disease prevention, care, and treatment, in problem-solving and public-speaking
• Well-being among Latino men
• Community members engage</td><td>Conclusion
This study provides preliminary evidence that this approach is feasible and appropriate for use with Latino men and can be effective in reaching men who might otherwise be difficult to reach.</td></tr>
<tr><td colspan="2">Methodology
• Qualitative life story interviews</td></tr>
<tr><td colspan="2">Sample
• 9 navegantes</td></tr>
</table>

16. Torpey KE, Kabaso ME, Mutale LN, Kamanga MK, Mwango AJ, Simpungwe J, et al. Adherence support workers: a way to address human resource constraints in antiretroviral treatment programs in the public health setting in Zambia. PLoS One. 2008;3(5):e2204.

**Setting**<br>Zambia	
**Context**<br>• Rural setting<br>• High HIV prevalence<br>• HIV stigma<br>• Health worker shortage: the rapid expansion of access to ART and the increasing patient load will put a strain on the existing fragile human resource base	
**Intervention characteristics**<br>• Peer to peer model. Adherence support workers (ASWs) are PLWHA, often on ART themselves<br>• Hosting by NGO<br>• ASWs provide 20 hours per week volunteering<br>• Facilities and community level<br>Support<br>• Standardized curriculum (10 days training) with practical attachment and supervision from health care workers. The training provides technical information on ART and adherence, plus additional modules on techniques for relationship building, counselling skills. The modules involve didactic sessions, role plays, group and individual exercises<br>• Stipends<br><br>Process of implementation<br>• ASWs provide empathetic and emotional support to patients (ASWs are able to remove fears, myths and misconception; patients feel encouraged). ASWs are perceived as a role models for demonstrating the benefits of treatment adherence	
**Mechanisms**<br>• ASW HIV personal status plus the training allow the ASWs to speak from a practical and personal standpoint giving them a sense of self efficacy<br>• ASW HIV personal status and being non-medical people contribute to a close relationship between ASW and patient	
**Outcomes**<br>• ASWs actively perform expected task<br>• Good quality of counselling<br>• Reduce patient waiting time and the workload of health care workers	**Conclusion**<br>Adherence counselling tasks can be shifted to lay cadres like ASWs without compromising the quality of counselling. Follow-up of clients by ASWs within the community is necessary to improve retention of clients on ART.
**Methodology**<br>• Mix (cohort, retrospective record review, focus group discussion, interviews)	
**Sample**<br>• 500 patients' records, interviews with 19 lay counsellors, 95 patients, 10 managers at health facility and focus group discussion with 16 health care workers	

17. Schneider H, Hlophe H, van Rensburg D. Community health workers and the response to HIV/AIDS in South Africa: tensions and prospects. Health Policy Plan. 2008;23:179-87.

**Setting**<br>South Africa
**Context**<br>• HIV stigma<br>• Large scale unemployment<br>• CHW volunteers integrated in the primary health care system<br>• Partnership between state and NGO<br>• NGOs are seen by the community as little more than organisations that disburse stipends<br>• CHWs overwhelmingly female, predominantly between the ages of 30 and 50 years<br><br><u>Link to task shifting strategies: context of CHWs</u><br>• Policies allow for a degree of ambiguity and interpretation (such as proposing remuneration of CHWs while not precluding ongoing recruitment of volunteers; being oriented to more traditional notions of CHWs (community-based generalist workers) whilst acknowledging the reality of more limited purpose CHWs)<br>• Step toward regularisation of stipend payments and formalisation of CHWs as employees (even though there were still problems with inconsistent and unreliable processes of payment). However, the state has deliberately avoided incorporating them into the civil service as formal employees. This has been made possible by the use of NGO intermediaries: CHWs were officially employed by NGOs in the province
**Intervention characteristics**<br>• Recruitment and selection calls for volunteers via community-based organisations and with the involvement of local health facility staff (seeking to recruit PLWHA).<br>• Task shifting from health workers to CHWs (reorganisation and expansion of job descriptions for CHWs)<br>• Ongoing training through provincially contracted NGOs<br>• Shift towards more multi-skilled and multi-purpose HIV/TB workers, even if not generalist CHWs<br>• Supplies such as gloves and kits for home-based care<br><br><u>Implementation process</u><br>• Nurses fear to delegate sensitive tasks such as HIV counselling and are concerned with losing their territory as professionals
**Mechanisms**<br>• The new expansion of role with further training triggers a growing sense of self-efficacy<br>• Hope for employment<br>• Participating in such an intervention triggers a feeling of fulfilling identity-related need (opportunities for personal growth, expressing religious identities of altruism and caring, development of new and not stigmatised personal identity, improvement in social status)<br>• Precariousness of employment without normal rights while being expected to work regular hours triggers a feeling of lack of recognition in the eyes of health care authorities, facilities and communities and contributes to dissatisfaction<br>• The hierarchical relationship with clinic nurses triggers a lack of trusting relationship between CHW and clinical staff and the sense of not belonging to the formal health team contributes to dissatisfaction
**Outcomes/conclusion**<br>• Despite the growing sense of self-efficacy, interviews with CHWs remained marked with high level of dissatisfaction<br>• Rewards, such as stipends and fulfilling identify-related needs played a key role in retaining CHWs
**Methodology**<br>• Qualitative
**Sample**<br>260 CHWs

18. Arem H, Nakyanjo N, Kagaayi J, Mulamba J, Nakigozi G, Serwadda D, et al. Peer health workers and AIDS care in Rakai, Uganda: a mixed methods operations research evaluation of a cluster-randomized trial. AIDS Patient Care STDS. 2011;25:719-24.

Setting
Uganda

Context
- Rural and low-resource setting
- Setting relatively atypical, mobile clinic approach
- ART and barrier to care: early ART-related difficulties such as stigma, side effects, technical understanding of how to take drug
- Later adherence barrier such as treatment fatigue, sense that they are cured, difficulty maintaining their drug schedule at work
- Health workforce crisis

Intervention characteristics
- Task shift through training selected patients as peer health workers (PHWs)
- Intervention by peers who are socio-culturally alike (PHW are present in the community, are knowledgeable about the community and know about long-term barriers to care)
- PHWs provided adherence monitoring and psychosocial support to fellow patients at clinic sites and during periodic home visits and assisted with triaging sick patients
- Supervision and ongoing feedback from clinical staff
- Appropriate incentives to compensate how PHW home visiting took time away from other gainful work

Implementation process
- Logistical support from the local health clinic (programmatic improvements were implemented during the study period besides the PHW intervention and resulted in lower than anticipated rates of virologic failures)

Mechanisms
- Training provides knowledge
- Intervention by peers was meant to trigger an aspirational adoption of the messages by patient and did not have any obvious deleterious effects on disclosure and confidentiality
- Trusting and close relationship among provider-patient: the relatedness between the patient and PHW led to both responsiveness and responsibility amongst CHW
- Possibility of being compensated with stipends may have motivated PHW to perform well and to be retained

Outcomes
- PHWs generally fulfilled their tasks and reduced the overall workload for clinical staff
- PHWs have good competences in AIDS/HIV counselling, in providing consistent information and psychosocial support and in combating stigma
- Appropriate stipends contributed to PHWs' high retention rate
- Inclement weather, absent patient and transportation challenges reduced PHW home visit

Conclusion
PHW positively impact adherence.

Methodology
- Mixed (qualitative: in-depth interviews, group discussions, direct observations and quantitative: survey of clinic staff/patient virologic outcome data, process data analysis)
- Cluster randomised trial

Sample
- 38 interviews (patients, staff and PHWs)
- 6 patient focus groups and 2 PHW focus groups
- 11 direct observations during PHW home visits

19. Chibanda D, Mesu P, Kajawu L, Cowan F, Araya R, Abas MA. Problem-solving therapy for depression and common mental disorders in Zimbabwe: piloting a task-shifting primary mental health care intervention in a population with a high prevalence of people living with HIV. BMC Public Health. 2011;11:828

**Setting**<br>Zimbabwe
**Context**<br>• Ethnic diversity<br>• Urban setting<br>• High prevalence of people living with HIV<br>• High unemployment with most residents relying on informal trading<br>• Lay health workers (LHW): female LHW are a respected group of primary health care providers whose roles include supporting people living with HIV and carrying out health promotion activities
**Intervention characteristics**<br>• Intervention theoretically closely linked to problem-solving therapy that includes an activity scheduling component with cultural local adaptation<br>• Home visit<br>• Training and refresher training<br>• Provision of locally adapted tools<br>• Peer and nurse-led supervision<br>• Incentives: linkage with two local income-generating projects
**Mechanisms**<br>• The training and the tools triggered a sense of self-efficacy<br>• The LHWs' position of trust in the community and the implementation of the intervention through an existing valued public health provider led to responsiveness amongst lay workers
**Outcomes/conclusion**<br>• It is feasible for lay workers to deliver this intervention for depression and common mental disorders and the lay workers were able to integrate the intervention into their routine work<br>• The intervention is efficacious in reducing psychological morbidity
**Methodology**<br>• Mix (qualitative group discussion and questionnaire for the lay workers to evaluate the intervention, quantitative survey for patient)<br>• Cross sectional study
**Sample**<br>• 10 lay workers, 320 patients

20. Elmardi KA, Malik EM, Abdelgadir T, Ali SH, Elsyed AH, Mudather MA, et al. Feasibility and acceptability of home-based management of malaria strategy adapted to Sudan's conditions using artemisinin-based combination therapy and rapid diagnostic test. Malar J. 2009;8:39.

Setting
Sudan

Context
- Rural and low-resource setting with famine, internal displacement and influx of refugees
- Existing problems associated with malaria treatment: accessibility to health services, expensive drugs and difficult to find, treatment on clinical bases
- Mortality due to malaria is high (low coverage and use of insecticide-treated bed nets)
- Effective malaria rapid diagnosis test (RDT)
- Volunteers tend to have low income, have a profession, are available in the area most of the time, acceptable by the community, have reading and writing ability, and are willing to work as volunteer

Intervention characteristics
- Home visits
- Clear positioning of the volunteers within community, such as establishment of a village health committee and development of opportunities for community feedback
- Involvement of patients, villagers in recruiting volunteers, supervising and supporting the project
- Training skills-based (involving problem solving, group work, exercises, demonstration and practical sessions) and refresher training
- Interpersonal communication methods and tools developed by the investigators and the volunteers themselves
- Sensitisation and advocacy for the communities to use the home-based management of malaria (HMM) services, where community leaders played a great role
- Small fees (during consultation)

Intervention implementation
- Supply, storage and distribution system of Artemisinin combination therapies (ACTs) and RDTs are satisfying.
- Community support and satisfaction with the project implementation/patient adherence to the programme (the service was easy, nearby and cheap). The selected malaria control assistants (MCAs) were accepted because of their honesty, accessibility, the way they dealt with their patients and knew what to do, and the way they gave time to their patients

Mechanisms
- Assurance that there is a system of back-up in case of difficulty (active and supportive village health committees)
- Volunteers are motivated because participation in the programme triggers a feeling of having a valuable spiritual and social role
- Volunteers are motivated because of community respect and support
- Volunteers felt that the financial gain was not enough to lead them to leave their original work. According to the author, this can be an important factor for the sustainability of the project

Outcomes
- Adherence to the project design and guidelines
- Expected tasks well performed
- The interpersonal communication methods and tools developed by the investigators and the MCAs themselves contributed to MCAs' good performance in initiation of health promotion and community mobilisation activities in their communities

Conclusion
MCAs prove their abilities to record and report cases by correctly using the format for registration and reporting. The project increased treatment-seeking behaviour and increased access to anti-malarial drugs. Although the project needs some adaptation, its general feasibility and acceptability was proved.

Methodology
- Mixed (quantitative: household survey of beneficiaries and records/report analysis of volunteers' work; qualitative: group discussion with community leaders and interviews with volunteers)
- Cross sectional study

Sample
- 892 beneficiaries, 4 group discussions with community leaders, 20 volunteers

21. Hoke TH, Wheeler SB, Lynd K, Green MS, Razafindravony BH, Rasamihajamanana E, et al. Community-based provision of injectable contraceptives in Madagascar: 'task shifting' to expand access to injectable contraceptives. Health Policy Plan. 2012;27:52-9.

Setting

Madagascar

Context

- Rural or extra urban setting
- Underserved area: cultural (knowledge about health is low, false beliefs), geographical and financial barrier to care
- Poor beneficiary population and unmet need for family planning (contraceptive prevalence is low and access to health services is poor)
- Demand for injectable contraceptives is high
- Overstretched health work force
- Rules and official policies for task shifting in place in Madagascar
- Community-based distribution (CBD) workers are literate

Intervention characteristics

- Intervention organised by the Ministry of Health and family planning supported by a NGO
- CBD worker recruitment and assessment: females were strongly preferred. Past success in attracting and retaining clients
- Prior to initiating injectable services provision, each CBD worker was formally reintroduced into his or her community in a small ceremony officiated over by the mayor.
- Delivering services in clients' home and in CBD workers' home
- Curriculum and job aids adapted to Madagascar context
- 3 days' skills-based certificated training including classroom instruction and practice of safe injection technique
- Supervision and follow-up (clinicians from the health centre and supervisors from supporting NGO)
- Incentives: modest profit from each dose
- Ministry of health and family planning reporting forms
- Provision of backpack with the Ministry of Health and family planning logo and well recognised logos of the NGO: initial supply of injectable contraceptives, free of charge
- Workers expected to travel monthly to their assigned health facility to submit a monthly activity report and obtain new supplies

Intervention implementation

- Travel to the health centres is impossible during certain times of the year (seasonal rains or other obstacles) and can trigger difficulties in obtaining commodities
- Obstacles preventing supervisors from meeting with CBD workers (long distances to reach the CBD worker, other work responsibilities, lack of travel resources, weather and lack of supervisor compensation)

Mechanisms

- CBD workers felt the training left them fully prepared to carry out their duties (learning a new skill, being able to serve more clients and being able to serve clients better)
- Consistent supply of injectable contraceptives can keep CBD workers motivated
- Streamlined reporting can motivate CBD workers to keep good records and not to feel over-burdened with administrative tasks

Outcomes

- CBD workers good and sustained competences
- Reduction of unmet need for contraception: attraction of clients for the delivery of injectable services
- High re-injection rate
- Large proportion of clients who were new family planning users
- Participation in the programme, learning new skills and meeting the need of the community contributed to job-related satisfaction and retention

Conclusion

CBD workers maintained quality standards as they offered this new service.

Challenges: missing supplies, long travel time to reach clients' homes and the health centres, modest compensation undermined the CBD workers' satisfaction.

Methodology

- Mixed (review of CBD workers' administrative records, structured interviews with CBD workers, supervisors and clients)
- Cross sectional study

Sample

61 CBD workers and 303 clients

22. Sanjana P, Torpey K, Schwarzwalder A, Simumba C, Kasonde P, Nyirenda L, et al. Task-shifting HIV counselling and testing services in Zambia: the role of lay counsellors. Hum Resour Health. 2009;7:44.

**Setting**<br>Zambia
**Context**<br>• HIV Stigma<br>• High HIV/AIDS prevalence<br>• Rapid expansion of access to ART<br>• Shortage of health workers and low ART coverage<br>• Rules and legal framework for task shifting in place
**Intervention characteristics**<br>• Services are provided two to three days per week in health facilities<br>• Lay counsellors are selected based on their ability to read and write in English, must reside within the facility catchment area, and have experience with the health facility for at least one year<br>• Skills-based training and certification using the standard national counselling and testing curriculum: two-week classroom component followed by a four-week supervised practical component<br>• Lay counsellors are integrated into the operation of their facilities<br>• Ongoing supervision by facility manager<br>• Stipends to cover travel expenses
**Mechanisms**<br>• Participation in the programme provides the ability for lay counsellors to help people and serve the community<br>• Training and supervision trigger a sense of self-confidence in their counselling skills<br>• Lay counsellors found their work rewarding<br>• Participation in the programme triggers a sense of influence/self-efficacy in lessening stigma and representing community role models. This reinforces a feeling of improvement in social status and a sense of relatedness with and accountability to the beneficiaries<br>• The lay counsellors have a deep sense of commitment to their role in the health facility and view themselves as professionals<br>• The feeling that a travel stipend is not enough compensation for services provided may push lay counsellors to find additional paid employment elsewhere and to leave the programme
**Outcomes/conclusion**<br>• 70 per cent of counselling provided by lay counsellors<br>• Lower error rate in medical records filled by lay counsellors than health care workers<br>• Lay counsellors are actively providing services<br>• Good quality of services<br>• Lay counsellors are acceptable providers and readily used by clients
**Methodology**<br>• Mixed (interviews, focus group discussions, review of two-years records pre and post deployment of CHWs)<br>• Cross sectional study
**Sample**<br>• 19 lay counsellors, 121 health workers, 1083 register entries, 260 CHWs

23. Cornish F, Campbell C. The social conditions for successful peer education: A comparison of two HIV prevention programmes runs by sex workers in India and South Africa. Am j community psychol. 2009;44:123–35.

India: The Sonagachi Project

**Setting**<br>Sonagachi, Kolkata
**Context**<br>• Marginalised, stigmatised and disempowered beneficiary population (female commercial sex workers from very deprived backgrounds, living in conditions of poverty and gender inequality, and at high risk of poor sexual health)<br>• Minimal physical infrastructure exists<br>• Relationships between sex workers are often characterised by competitiveness and isolation but with some form of solidarity<br>• Established red light district with hierarchical (and exploitative) social organisation (existing legitimacy of social order to build upon)<br>• Familiarity with themes of democracy and workers' movements<br>• Sex workers consider themselves as citizens with legitimate demands and the beginnings of confidence in the ability of their group to effect change
**Intervention characteristics**<br>• Peer education led by sex workers as an approach to HIV prevention<br>• Community engagement and consultation/engagement of wide range of powerful stakeholders<br>• Wider community development (addresses the local social problems which sex workers face)<br>• Training in health-related information and communication skills, intensive support of peer educators in development of leadership experience and skills<br>• Promotion of critical thinking about the stigmatisation of sex workers<br>• Supervision, problem solving and ongoing field support<br>• Monetary incentives: small salary for their part-time work<br>• Committees of sex workers mediate in local conflicts<br>• Sex workers are well represented on decision making committees<br><br><u>Project implementation</u><br>• Project avoided local power struggles<br>• Established strong and united group of empowered and mobilised peer educators
**Mechanisms**<br>• Due to the lack of other sources of esteem or resources, peer educators want to join the programme in the hope of benefiting individually from it (consequent solidarity and support that the project has to offer)<br>• Financial incentives and opportunities to secure their rights (wider community development) may motivate peer educators to stay<br>• Participation in the programme triggers a sense of improving social status and of developing a positive social identity<br>• Local sense of ownership/greater control in their lives<br>• Feeling of self-respect and confidence
**Outcomes/conclusion**<br>• Peer educators' long-term commitment to the project (the small salary for their part-time work, which, together with the positive social identity of being a health worker, makes sex workers keen to get the jobs and to keep them)<br>• Peer educators are credible source of communication<br>• Increased condom use and decreased levels of sexually transmitted infections
**Methodology**<br>• Ethnographic case studies<br>• Qualitative interviews and group discussions with participants and key informants; review of documents and participant observation
**Sample**<br>19 participants, 110 key informants

Africa: The Summertown Project

Setting

Summertown, South Africa

Context

- Marginalised, stigmatised and disempowered beneficiary population (female commercial sex workers from very deprived backgrounds, living in conditions of poverty and gender inequality, and at high risk of poor sexual health)
- Set of isolated and poverty-stricken illegal squatter camps
- No infrastructure, insecure shack settlements
- No stable political support: little evidence locally that poor women can have power
- Relationships between sex workers are often characterised by competitiveness and jealousy
- No stable social organisation of the communities (there is a high turnover among the inhabitants and thus little opportunity for stable social relationships or supportive norms to develop)

Intervention characteristics

- Peer education led by sex workers and migrant mineworkers as an approach to HIV prevention
- An inspired nursing sister was employed as outreach coordinator to run the sex worker peer education programme
- Multi-stakeholder management: management committee with a range of powerful stakeholders
- Mobilisation of a group of women to form a peer education group
- Sex workers are not involved in decision-making and are not represented in the committee
- Peer educators were trained in participatory health promotion methods and in organisational skills for monitoring the quality of peer education and conducting meetings
- Peer educators have weekly meetings with outreach coordinator
- Non-financial incentives (tee-shirts, condoms, training, opportunities to travel and social status)

<u>Project implementation</u>

- Peer educators are embroiled in local power structure and are not respected by other sex workers
- The stakeholder committee did not become a cohesive and active group
- No support from the directors of the project to solve social issues

Mechanisms

- Due to the lack of other sources of esteem or resources, peer educators want to join the programme in the hope of benefiting individually from it (consequent solidarity and support that the project has to offer)
- No feeling of self-respect and confidence
- No confidence in the ability to change health problems

Outcomes

- No credible source of communication
- Peer educator group insufficiently organised to maintain their health promotion activities
- Failed in its aims to mobilise an organised local response
- No impact on levels of condom use or HIV/AIDS, and was not sustainable

Conclusion

The peer educators' role remained a difficult one, as their peers were often suspicious of their motives, or simply had insufficient control over their own sexual encounters to be able to put the peer educators' advice into practice.

Methodology

- Ethnographic case studies
- Qualitative in depth interviews with participants and interviews with key informants
- Review of documents and participant observation

Sample

- 20 participants
